# Inhibition of RNase to Attenuate Fungal‐Manipulated Rhizosphere Microbiome and Diseases

**DOI:** 10.1002/advs.202503146

**Published:** 2025-08-07

**Authors:** Bo Yang, Sen Yang, Xiaomi Wang, Yuanwei Zhang, Yao Zhao, Menghuan Tao, Jinyi Zhu, Wanxin Zhang, Yansu Wang, Kaixuan Duan, Yan Wang, Wenwu Ye, Zhenfei Guo, Yuanchao Wang

**Affiliations:** ^1^ College of Grassland Science Nanjing Agricultural University Nanjing 210095 China; ^2^ Department of Plant Pathology Nanjing Agricultural University Nanjing 210095 China; ^3^ Key Laboratory of Soil Environment and Pollution Remediation Institute of Soil Science Chinese Academy of Sciences Nanjing 210008 China; ^4^ University of Chinese Academy of Sciences Nanjing 211135 China; ^5^ College of Life Sciences Nanjing Normal University Nanjing 210023 China; ^6^ Institute for Plant Sciences University of Cologne Cluster of Excellence on Plant Sciences (CEPLAS) 50674 Cologne Germany; ^7^ Institute of Fundamental and Frontier Sciences University of Electronic Science and Technology of China Chengdu 610054 China

**Keywords:** effector, fungal pathogen, GMP, microbiome, RNase, soybean disease

## Abstract

Soil‐borne pathogens must cross the barriers of plant‐associated microbiota to cause disease. Phytopathogens utilize effector proteins to manipulate host immunity and promote niche colonization. Identifying core effectors involved in both pathogen‐microbiota and pathogen‐host interactions is vital for understanding pathogenic mechanisms and designing targeted chemical compounds for disease prevention. Here, this work shows that the soil‐borne pathogen *Fusarium graminearum* can manipulate the plant‐associated rhizosphere microbiome through the virulence effector Fg12, which encodes a fungal‐specific ribonuclease (RNase). Fg12 is widely distributed among various fungal pathogens, and its antibacterial function relies on RNase activity. Several Fg12‐inhibited bacterial strains, both individually and in synthetic communities (Syncoms), can alleviate *Fusarium* infection in soybean and alfalfa plants. Moreover, this work employs structural modeling, molecular docking, and in vitro enzymatic assays to demonstrate that guanosine monophosphate (GMP) functions as an effective chemical inhibitor of Fg12. Notably, GMP efficiently inhibits the antibacterial activity of Fg12 and alleviates disease symptoms in soybean and alfalfa. In conclusion, this work demonstrates that a virulence effector of a fungal phytopathogen can interfere with the host microbiome and propose GMP as a promising RNase inhibitor for attenuating plant fungal diseases.

## Graphical abstract

Fusarium graminearum uses its virulence effector Fg12, a fungal RNase, to manipulate the host rhizosphere microbiome and promote infection. Guanosine monophosphate (GMP) blocks Fg12's RNase activity. This reveals a key pathogen‐microbiota interaction mechanism and proposes GMP as a targeted strategy to disarm this core fungal effector, offering a promising approach to reduce soil‐borne diseases in crops like soybean and alfalfa.

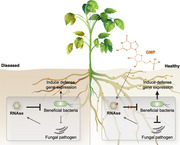

## 1 Introduction

In natural environments, complex microbial communities associate with host plants, forming a stable microbiota.^[^ ^1^ ^]^ Plant‐associated microbial communities not only promote host growth and development but also enhance adaptation to abiotic and biotic stresses.^[^ ^2^, ^3^ ^]^ The balance between plants and their microbiota can be disturbed under certain conditions, resulting in microbial community alterations termed dysbiosis, commonly associated with disease.^[^ ^4^ ^]^ The plant immune system plays a crucial role in maintaining and controlling microbiota homeostasis to prevent dysbiosis.^[^ ^5^ ^]^ For instance, activation of plant immunity leads to the release of root exudates that stimulate, enrich, and support the growth of soil microbes, acting as the first line of defense against soil‐borne pathogens.^[^ ^6^ ^]^ From the perspective of pathogens, the ability to disrupt host‐associated microbiota is a critical first step for successful host infection.^[^ ^7^ ^]^ Phytopathogens have evolved strategies to modulate host‐associated microbiota, thereby facilitating plant colonization. For example, *Verticillium dahliae* secretes effectors with selective antimicrobial activity against specific bacteria or fungi to manipulate microbiota and facilitate colonization.^[^ ^8^, ^9^, ^10^ ^]^ Opportunistic *Xanthomonas* pathogens secrete T2SS‐dependent enzymes to enhance their competitiveness in the microbiota.^[^ ^4^ ^]^ Conversely, beneficial bacteria associated with plants or insects can secrete antimicrobial effectors to antagonize surrounding bacterial or fungal pathogens.^[^ ^11^, ^12^ ^]^ These findings add complexity to the tripartite interactions resulting in plant disease. Unveiling how pathogens modulate host microbiota is crucial for developing disease‐protective microbial communities resistant to pathogen manipulation.

Initially, effectors were regarded as pathogen‐specific proteins that facilitate infection during plant‐pathogen interactions.^[^ ^13^ ^]^ Recent research has unveiled the interactions between microbes and the plant immune system, elucidating the molecular mechanisms by which effectors activate or evade immunity.^[^ ^13^, ^14^ ^]^ Several studies suggest that pathogen virulence effectors can be potential targets for developing anti‐disease agents.^[^ ^15^, ^16^, ^17^ ^]^ Considering that effectors are crucial for soil‐borne pathogens to survive in soil and navigate host root microbiota, identifying core effectors involved in both pathogen‐microbiota and pathogen‐host interactions is vital for understanding pathogenic mechanisms and designing targeted chemical compounds for disease prevention.

*Fusarium* is a genus of filamentous fungi encompassing many agronomically important plant pathogens that cause root rots, blights, wilts, and cankers in a wide range of crops worldwide.^[^ ^18^ ^]^ For instance, *F. graminearum* causes several devastating diseases, including *Fusarium* head blight (FHB), crown rot in barley and wheat, ear and stalk rot in corn, and soybean root rot.^[^ ^19^, ^20^, ^21^, ^22^ ^]^ It is ranked among the top 10 fungal pathogens of plants.^[^ ^23^ ^]^ As a soil‐borne pathogen, *F. graminearum* often co‐infects with other fungi or oomycetes.^[^ ^23^ ^]^ Through proteomic analysis and functional verification, we previously identified that the *F. graminearum*‐secreted effector protein Fg12 encodes a functional fungal ribonuclease (RNase). The Fg12 gene is significantly upregulated during soybean infection, targets host cells to induce cell death, and contributes to *F. graminearum* virulence.^[^ ^22^ ^]^ Similarly, the secreted RNase SRE1 from *Setosphaeria turcica* contributes to its virulence.^[^ ^24^ ^]^ Evidence suggests that the secreted RNase ZT6 from *Zymoseptoria tritici* may function in antimicrobial competition and niche protection.^[^ ^25^ ^]^ RNase T1 from *Aspergillus oryzae* is the most thoroughly studied member of fungal RNases. Its crystalline structure in complexes with various inhibitors (2'‐guanosine monophosphate, 2'‐GMP; 3'‐guanosine monophosphate, 3'‐GMP; 5'‐guanosine monophosphate, 5'‐GMP) has been determined or predicted, and its mechanism of action has been studied in detail.^[^ ^26^, ^27^, ^28^ ^]^

Here, we describe the additional function of the *F. graminearum* effector Fg12, which not only targets plant cells but also exhibits antibacterial activity, facilitating plant colonization through the direct suppression of *Pseudomonas* and *Bacillus* within the host microbiota. Additionally, we found that the compound 5'‐GMP targets Fg12 and inhibits its RNase activity, effectively managing fungal diseases. Our findings open up new strategies for both biological and chemical control of fungal pathogens.

## 2 Results

### 2.1 Fg12 Contributes to the Colonization of Fusarium Graminearum in Soil

In our previous study, we identified the *Fg12* gene encoding a secreted RNase as being highly upregulated during the early stages of *F. graminearum* infection.^[^ ^22^ ^]^ Interestingly, culturing *F. graminearum* in a simulated soil environment, consisting of soil extract at varying concentrations mixed with PDB medium, significantly upregulated the expression of the *Fg12* gene compared to cultivation in PDB medium alone. Moreover, *Fg12* expression increased proportionally with higher concentrations of soil extract (Figure S1A, Supporting Information). Next, we cultured wild‐type (WT) *F. graminearum* and ΔFg12 strains in pure PDB or PDB supplemented with 75% soil extract to collect their secreted proteins (SPs) and analyzed for RNase activity. Both strains exhibited detectable RNase activity in pure PDB, with ΔFg12 showing significantly reduced activity compared to WT (Figure S1B, Supporting Information). Cultures supplemented with soil extract exhibited higher RNase activity than pure PDB (Figure S1B, Supporting Information). Notably, ΔFg12 cultured in soil extract medium displayed substantially reduced activity compared to WT under identical conditions (Figure S1B, Supporting Information). These results suggest that Fg12 contributes not only to virulence during host infection but also plays a crucial role in soil colonization.

**Figure 1:**
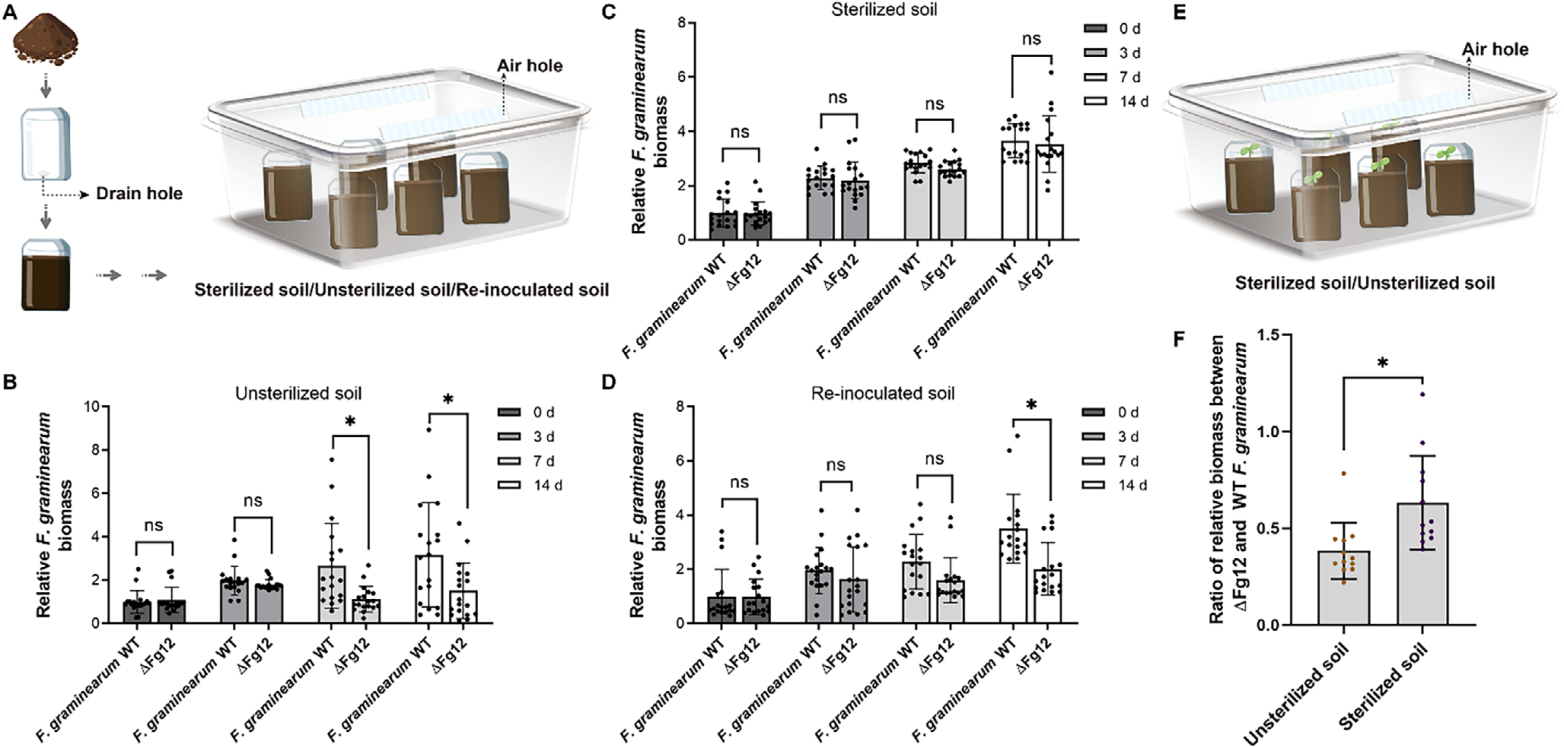
Fg12 promotes *Fusarium graminearum* colonization in soil. A) Schematic of the gnotobiotic soil colonization system. Soil treatments were added to three‐holed plant tissue culture flasks, sealed in microboxes with vented lids, and incubated in growth chambers. B) *F. graminearum* biomass in soil over 14 days post‐inoculation (wild‐type vs. Fg12 mutant), quantified by real‐time PCR (unpaired *t*‐test, N = 18). Asterisks denote significant reductions in mutant colonization (*p* < 0.05). C) No colonization difference between strains in sterile soil (unpaired *t‐*test, N = 18). D) Colonization assay in sterilized soil amended with 20% fresh soil (unpaired *t*‐test, N = 18). E) Gnotobiotic soybean growth system (modified from panel A). F) *F. graminearum* biomass in soybean roots. Ratios of Fg12 mutant‐to‐wild‐type biomass under different conditions were compared (unpaired *t*‐test, N = 12).

In order to further reveal the mechanism of Fg12 for *F. graminearum* colonization in soil, we inoculated *F. graminearum* WT and Fg12‐knockout mutant (ΔFg12) into soil in a gnotobiotic system, respectively (**Figure** **1**A), and measured fungal biomass at various time points. The results demonstrated that in unsterilized treatment group samples collected on days 7 and 14, the ΔFg12 mutant exhibited significantly lower soil colonization levels compared to the WT strain, confirming the essential role of Fg12 in soil colonization (Figure 1B). Notably, when the soil was sterilized, the colonization difference between the WT and ΔFg12 mutant was no longer significant (Figure 1C). However, supplementation with fresh soil restored the fitness advantage conferred by Fg12 to *F. graminearum* (Figure 1D), suggesting that the Fg12‐mediated colonization advantage is dependent on the soil microbiota. Additionally, we compared the colonization biomass ratios of the ΔFg12 mutant and WT *F. graminearum* in soybean roots under sterile and non‐sterile conditions by using a gnotobiotic plant growth system (Figure 1E).^[^ ^29^ ^]^ The results showed a significant difference in the colonization ratio under sterilized versus non‐sterilized conditions (Figure 1F), indicating that the virulence function of Fg12 is closely linked to interactions with soil microbes.

### 2.2 *F. graminearum* Fg12 Affects Bacteriome

**Figure 2:**
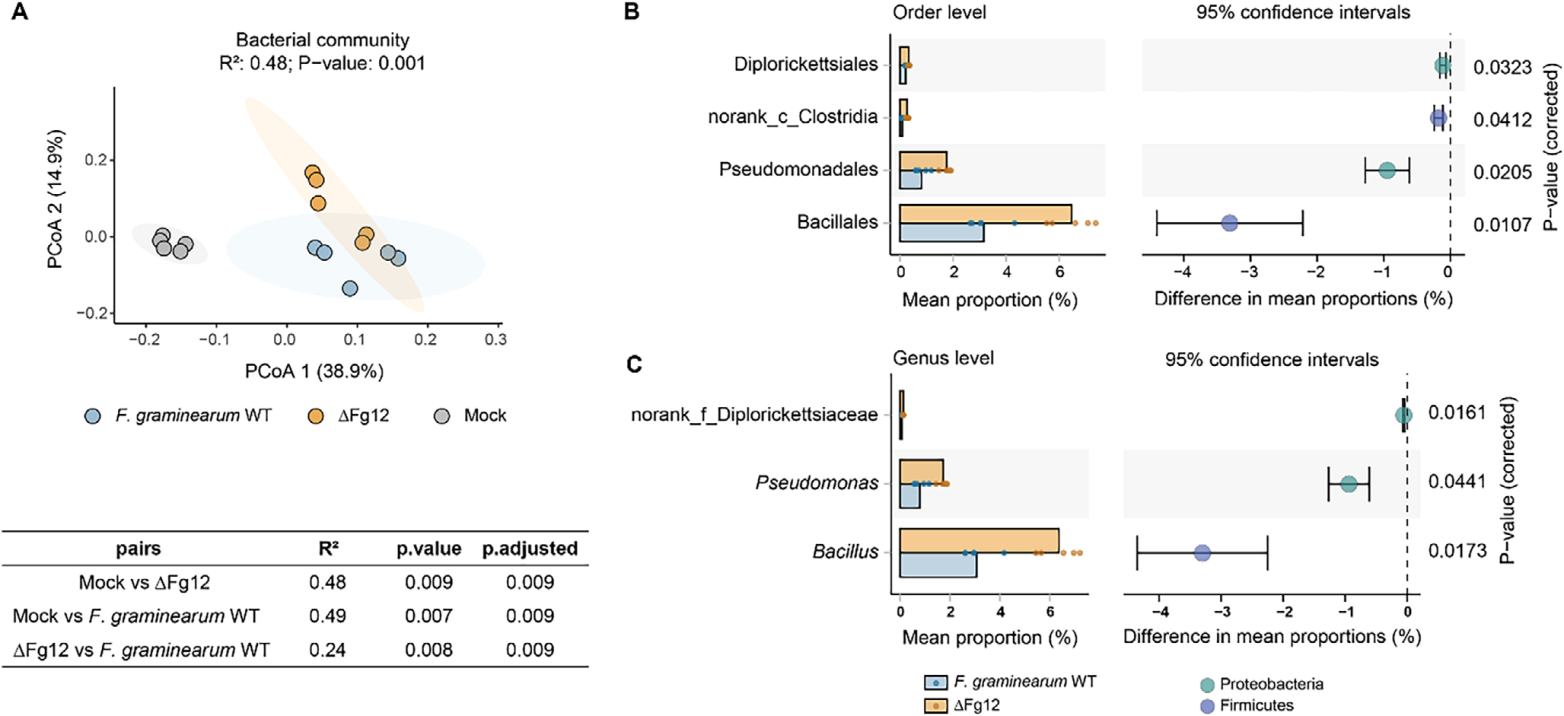
*Fusarium graminearum* Fg12 affects bacteriome. A) Principal coordinate analysis based on Bray–Curtis dissimilarities reveals separation of root bacteriome compositions 14 days post‐inoculation with wild‐type *F. graminearum* and an Fg12 deletion mutant (PERMANOVA, *p* < 0.01, N  =  5). Pairwise PERMANOVA results are displayed below each PCoA. Roots with rhizosphere soil from three soybean plants were pooled to form a single biological replicate. B) Differential abundance analysis of bacterial orders through pairwise comparison between root microbiomes colonized by wild‐type *F. graminearum* and a Fg12 deletion mutant (wilcox.test, *p* < 0.05). The mean proportion of differentially abundant taxa is indicated as a percentage of the total bacterial community in the corresponding root bacteriome. C) *Bacillus* and *Pseudomonas* are repressed by Fg12. Columns represent bacterial genera with increased abundance in root bacteriome on colonization by the Fg12 deletion mutant when compared with wild‐type *F. graminearum*.

To determine whether Fg12 secretion by *F. graminearum* impacts the soybean rhizosphere microbiome composition, we performed bacterial and fungal community analyses based on 16S and ITS ribosomal DNA profiling. Inoculation with *F. graminearum* WT or ΔFg12 mutant significantly reduced the total microbial α‐diversity in soybean roots compared to uninoculated controls (Figure S2A,B, Supporting Information). However, there were no significant difference in bacterial or fungal α‐diversity between *F. graminearum* WT and ΔFg12 mutant (Figure S2A,B, Supporting Information). Colonization by either *F. graminearum* WT or ΔFg12 mutant significantly altered the overall composition of both bacterial and fungal phyla (Figure S3A,B, Supporting Information). Notably, principal coordinate analysis based on Bray–Curtis dissimilarities (β‐diversity) revealed a clear separation of bacterial communities (**Figure** **2**A, permutational multivariate analysis of variance, PERMANOVA, *P* < 0.009) and fungal communities (Figure S4, Supporting Information, PERMANOVA, *P* < 0.018). To further explore which microbes are affected by Fg12, pairwise bacterial or fungal order comparisons in colonization by *F. graminearum* WT and the ΔFg12 mutant were performed. The results showed that Diplorickettsiales, taxa from class Clostridia, Pseudomonadales and Bacillales were the orders significantly suppressed in the microbiota colonized by *F. graminearum* WT secreting Fg12 (Figure 2B, Table S1, Supporting Information, *P* < 0.05). At genus level, the abundance of taxa belonging to family Diplorickettsiaceae, *Pseudomonas*, and *Bacillus* decreased significantly in the *F. graminearum* WT group compared to the ΔFg12 mutant group (Figure 2C, Table S2, Supporting Information, *P* < 0.05). We selected the genera *Pseudomonas* and *Bacillus* for further analysis, as norank_f_Diplorickettsiaceae cannot be classified at lower taxonomic ranks (where “norank” denotes that a specific taxonomic unit cannot be assigned to any recognized taxonomic rank within the reference database utilized for annotation). In addition, although β‐diversity analysis revealed significant differences in fungal communities, no significant genus‐ or order‐level differences were detected in subsequent analysis (Tables S3 and S4, Supporting Information).

### 2.3 Fg12 Selectively Inhibits In Vitro Growth of Soybean‐Associated Bacterial Isolates

To further investigate how Fg12 affects soybean‐associated *Pseudomonas* and *Bacillus*, we first isolated and identified bacterial strains from soybean roots inoculated with *F. graminearum* WT or the ΔFg12 mutant, focusing subsequent analysis on *Pseudomonas* and *Bacillus*. In total, 19 strains of *Bacillus* and 18 strains of *Pseudomonas* were isolated from the soybean roots, and these bacteria were identified as *B. safensis*, *B. velezensis*, *B. sp*., *P. putida*, *P. fluorescens*, and *P. sp*. (Table S5, Supporting Information). *Paenibacillus polymyxa* and some other bacteria were also identified (Table S5, Supporting Information).

Next, we produced and purified recombinant Fg12 protein using a heterologous expression system in *Escherichia coli*. As reported in our previous study,^[^ ^22^ ^]^ recombinant expression of Fg12 in *E. coli* yielded remarkably low protein levels, with a protein yield of 0.08 mg L^−1^. In contrast, the Fg12‐5A mutant, which was engineered through alanine substitution at all five RNase catalytic residues (Y64A, H66A, E84A, R102A, E117A), achieved a fourfold higher yield (0.32 mg L^−1^) under identical induction conditions (Figure S5, Supporting Information). Before isolating rhizosphere bacteria of soybean, we first carried out experiments with the well‐characterized *B. subtilis* strain OKB105, which has been reported in prior research.^[^ ^30^ ^]^ A concentration gradient assay identified the minimum inhibitory concentration (MIC) of Fg12 as 10 µM (Figure S6A, Supporting Information). The inhibition of Fg12 against *B. subtilis* was also verified by a halo test (Figure S6B,C, Supporting Information) and a *B. subtilis* Fg12‐secretion test (Figure S6D, Supporting Information).

**Figure 3:**
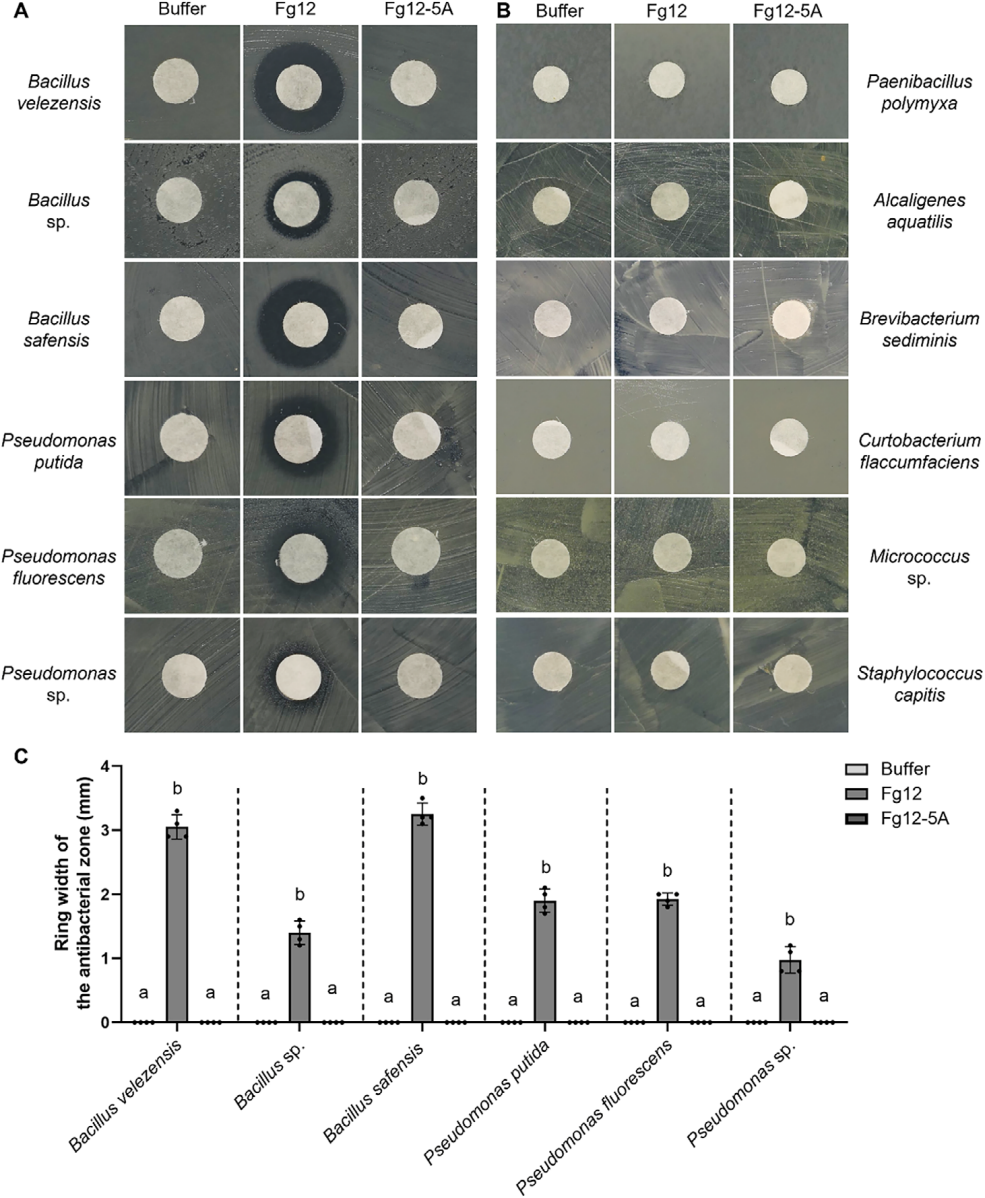
Fg12 selectively inhibits in vitro growth of soybean‐associated bacteria. A,B) Disk diffusion assay of Fg12 antimicrobial activity. Inhibition zones of soybean‐associated bacterial isolates treated with Fg12 (10µM), buffer control, or Fg12‐5A protein (10µM). Bacterial plates prepared with OD_600_ = 0.05. Images were captured 16 h post‐inoculation. C) Antibacterial zone width of *Bacillus* and *Pseudomonas* treated with Fg12, buffer, or Fg12‐5A measured 16 h post‐inoculation. Data represent the mean ± SD of four replicates. Different lowercase letters indicate significant differences (one‐way ANOVA, *p* < 0.05).

To test whether the suppression of *Pseudomonas* and *Bacillus* is a direct consequence of the antimicrobial activity of Fg12, we incubated *B. safensis*, *B. velezensis*, *B. sp*., *P. putida*, *P. fluorescens*, and *P. sp*. with 10 µM of Fg12 or Fg12‐5A and monitored their growth in vitro. *P. polymyxa*, *Alcaligenes aquatilis*, *Curtobacterium flaccumfaciens*, *Micrococcus* sp., *Staphylococcus capitis* and *Brevibacterium sediminis* served as negative controls. Interestingly, Fg12 inhibited the growth of *B. safensis*, *B. velezensis*, *B. sp., P. putida*, *P. fluorescens* and *P. sp*., whereas no growth inhibition was observed with Fg12‐5A (**Figure** **3A,C**;Figure S7A–F, Supporting Information), indicating that the RNase activity of Fg12 is important for its antibacterial effect. On the other hand, the growth of *P. polymyxa*, *Alcaligenes aquatilis*, *Curtobacterium flaccumfaciens*, *Micrococcus* sp., *Staphylococcus capitis* and *Brevibacterium sediminis* was not inhibited by either Fg12 or Fg12‐5A (Figure 3B; Figure S7G–L, Supporting Information), suggesting that *Pseudomonas* and *Bacillus* are genuine targets of Fg12 in soybean rhizosphere. Importantly, to assess functional relevance, we tested the antimicrobial activity of WT or ΔFg12 secreted proteins against *B. velezensis* and *P. putida*. WT‐secreted proteins from soil extract cultures formed distinct inhibition zones, while ΔFg12‐secreted proteins showed no inhibition (Figure S8A,B, Supporting Information). These findings confirm the antimicrobial function of Fg12, supporting its hypothesized role in suppressing competitors during rhizosphere colonization.

Additionally, Fg12 homologs were purified from *F. solani* (Fs12), *F. poae* (Fp12), *F. equiseti* (Fe12), and *Magnaporthe oryzae* (Mo12). These proteins exhibited inhibitory effects on bacterial and RNase activities similar to those of Fg12 (Figure S9A–D, Supporting Information). Fg12 was also tested for antifungal and anti‐*Phytophthora* activities. Treatment of *F. graminearum*, *Aspergillus japonicus*, *Gliocladium roseum, Trichoderma harzianum* and *P. sojae* with Fg12 or Fg12‐5A showed no significant growth inhibition effect (Figure S10, Supporting Information).

### 2.4 Fg12 is Widely Distributed among Different Fungi

**Figure 4:**
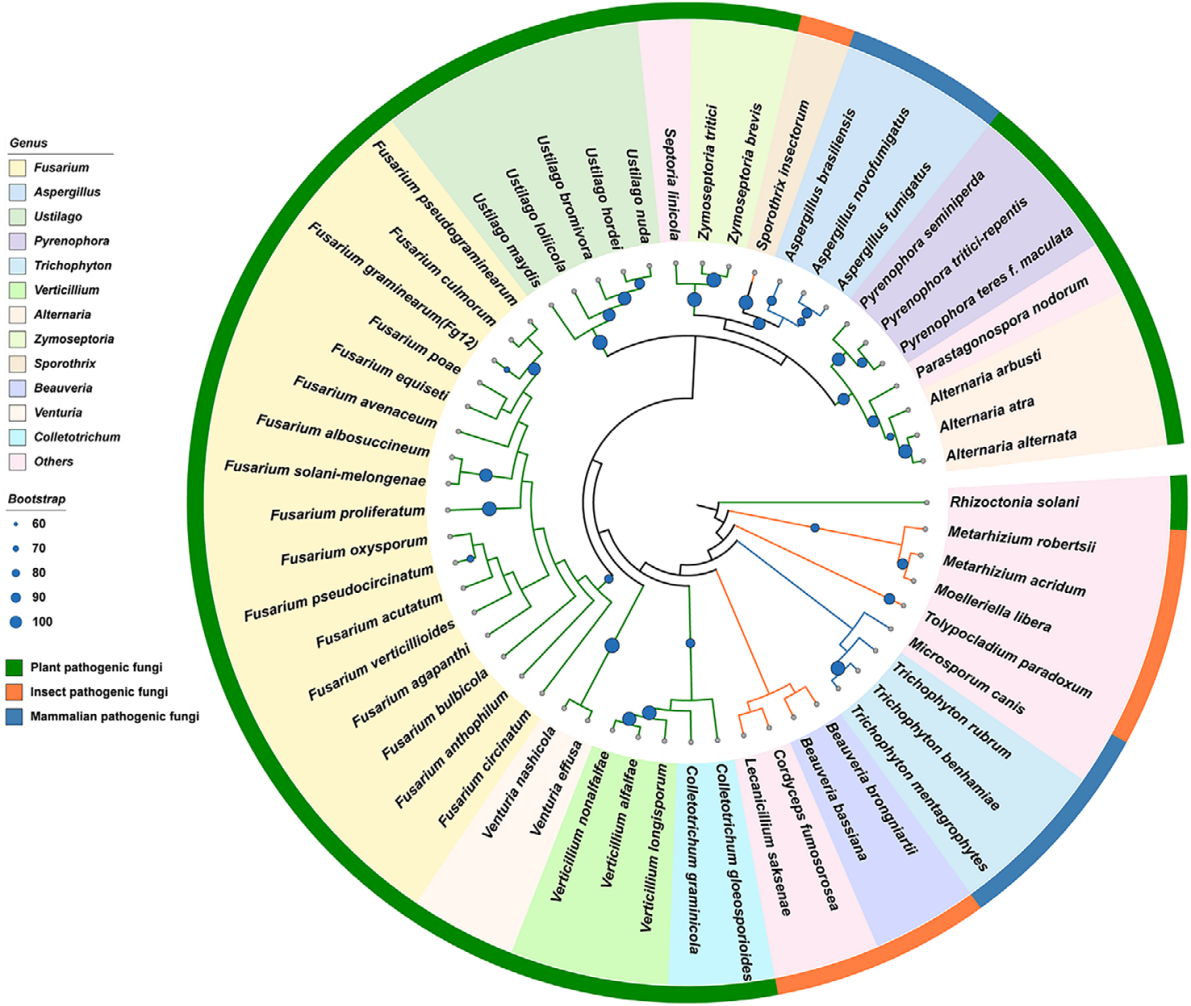
Fg12 is widely distributed among different fungi. A phylogenetic tree of selected Fg12 homologs reconstructed by RAxML is shown. For these RNase families, we performed multiple sequence alignments and maximum likelihood (ML) tree estimation. Sequence alignment was performed in MAFFT with default settings. TrimAl was used to trim the alignment to eliminate poorly aligned regions. The species tree was estimated in RAxML using the standard algorithm. Visualization of the tree was performed with iTOL.

We previously identified the effector Fg12 as a conserved secreted protein in different *Fusarium* species.^[^ ^22^ ^]^ Through further analysis of different pathogenic fungi of plants, insects or animals, we found that Fg12 homologs are conserved across a wide range of pathogenic fungi belonging to Ascomycota, such as the genus of *Fusarium*, *Aspergillus*, *Ustilago*, *Pyrenophora*, *Trichophyton*, *Verticillium*, *Alternaria*, *Zymoseptoria*, *Sporothrix*, *Beauveria*, *Venturia*, and *Colletotrichum* (**Figure** **4**). Sequence alignment showed that these homologs are conserved in the RNase functional domain (Figure S11, Supporting Information). Most of these homologs were predicted to be effectors (Table S6, Supporting Information), and nearly all predicted to contain signal peptides (Table S7, Supporting Information). This suggests that Fg12 is an evolutionarily ancient effector, and its broad conservation implies that Fg12 is a potential core effector across different fungi. Additionally, we also identified 336 putative Fg12 homologs in the NCBI database (Blast E value: 1e‐5) (Figure S12, Supporting Information), further supporting the widespread distribution of Fg12 across fungal lineages of Ascomycota and Basidiomycota.

### 2.5 Bacillus and Pseudomonas Mitigate Fusarium Root Rot Symptoms via Direct Antagonism and Induction of Plant Immune Responses

**Figure 5:**
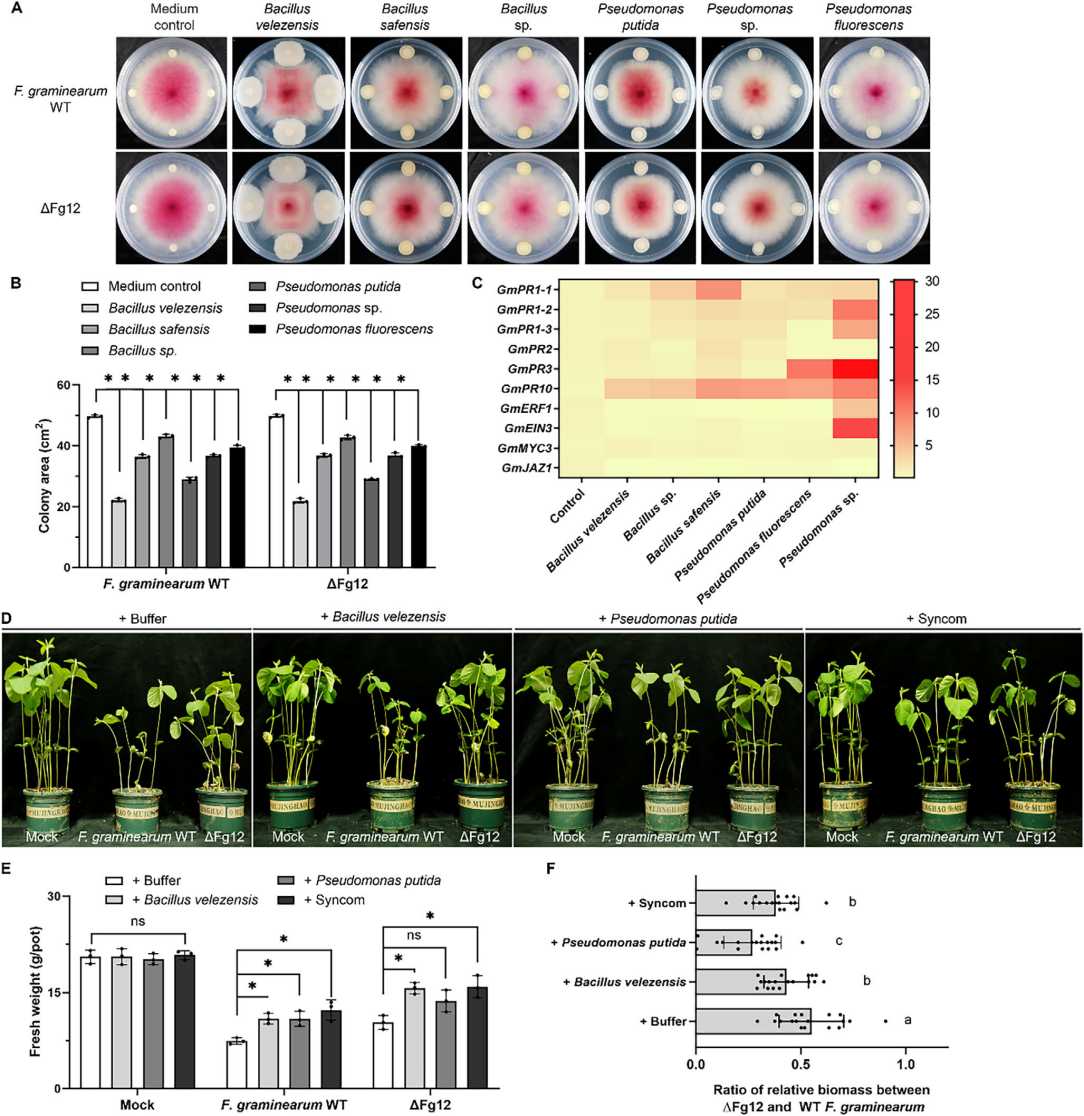
*Bacillus* and *Pseudomonas* mitigate *Fusarium* root rot symptoms in soybean. A) Antagonistic activity of plant‐associated *Bacillus* and *Pseudomonas* strains against *Fusarium graminearum* wild‐type (WT) and ΔFg12. *F. graminearum* PDA plugs were centered on plates, surrounded by sterile filter paper soaked in bacterial suspensions (test) or 10 mM MgSO_4_ (control). Images captured after 4‐day incubation at 25 °C. B) Colony area of *F. graminearum* WT and ΔFg12 co‐cultured with bacteria. Data represent mean ± SD (unpaired *t‐*test, N = 3). Asterisks indicate significant differences versus control (*p* < 0.05). C) Induction of soybean defense genes by *Bacillus* and *Pseudomonas*. qRT‐PCR analysis of defense‐related gene expression in roots 12 h post‐treatment with bacterial suspensions (OD_600_ = 0.01). Data normalized to *GmCYP2* and expressed as fold changes relative to water‐treated controls (three independent replicates). D) Phenotypes of soybean plants (cv. Hefeng47) 14 days post‐inoculation with *F. graminearum* WT, ΔFg12, or mock (V8 medium). Surface‐sterilized seeds were grown in soil (30% sterilized vermiculite) amended with fungal mycelium, followed by treatment with bacterial suspensions (OD_600_ = 0.1) or 10 mM MgSO_4_. Synthetic communities (syncom) were prepared by pooling six bacterial strains adjusted to equal OD_600_. Each pot contained 10 seeds (three replicates). E) Fresh biomass and F) *F. graminearum* biomass in soybean rhizosphere 14 days post‐sowing. Asterisks denote significant differences (unpaired *t*‐test: N = 3 for E; N = 18 for F; *p* < 0.05).

Based on microbiome analyses and in vitro experiments demonstrating that Fg12 secreted by *Fusarium* strongly inhibits *Bacillus* and *Pseudomonas*, we hypothesized that these bacteria may act as antagonists and negatively influence *F. graminearum* growth in the absence of Fg12. First, we evaluated the effects of different strains of *Bacillus* and *Pseudomonas* on the growth of *F. graminearum* on PDA plates, and observed that these strains exhibited varying degrees of inhibition against *F. graminearum* growth (**Figure** **5**A). Among them, *B. velezensis* and *P. putida* exhibited stronger antifungal activity against *F. graminearum*. Specifically, *B. velezensis* reduced the colony areas of the *F. graminearum* WT and ΔFg12 mutant by 55.52% and 56.03%, respectively, while *P. putida* decreased them by 41.78% and 41.76%, respectively (Figure 5A,B). We found *P. polymyxa*, which was not inhibited by Fg12, was able to antagonize *F. graminearum* (Figure S13A,B, Supporting Information). Second, inoculating soybeans with *Bacillus* or *Pseudomonas* isolate solutions individually, or with synthetic community (syncom, the mixture of 6 individual bacterial strains) of these isolates, had no adverse effects on soybean survival or growth (Figure S14, Supporting Information). Furthermore, to determine whether these bacteria could induce soybean defense responses, we examined the effects of bacterial treatment (OD_600_ = 0.01) on the expression of defense response‐related genes *PR1*, *PR2*, *PR3*, *PR10*, *ERF1*, *MYC3*, and *JAZ1* in soybean roots. The results showed that although the expression of individual defense‐related genes varied depending on the bacterial treatment, most of these bacteria could activate the immune response of soybean to different degrees (Figure 5C).

Next, we selected *B. velezensis* and *P. putida*, as well as a syncom, and analyzed their biocontrol effects on the occurrence of root rot after *F. graminearum* WT or ΔFg12 mutant inoculation in soybeans. The results demonstrated that under *F. graminearum* WT inoculation, both individual applications of *B. velezensis* and *P. putida*, as well as syncom, significantly enhanced soybean survival rate and biomass compared to the buffer control treatment. Specifically, *B. velezensis*, *P. putida*, and syncom inoculations increased soybean fresh weight by 46.86%, 46.51%, and 64.81%, respectively (Figures 5D,E; Figure S15, Supporting Information). To validate the inhibitory effect of Fg12 on bacteria under soil conditions, we employed a sterilized soil system to eliminate interference from native soil microbiota. *F. graminearum* WT or ΔFg12 mutant was inoculated, followed by supplementation with *B. velezensis* or *P. putida*. Bacterial viability was quantified through plate counting. Results revealed that ΔFg12‐inoculated groups showed a 69.9% increase in *B. velezensis* colonization and a 29% elevation in *P. putida* survival compared to *F. graminearum* WT treatments (Figure S16, Supporting Information).

To further clarify the contribution of Fg12 to ecological fitness during microbial competition, we compared the colonization biomass ratios of *ΔFg12* mutant and *F. graminearum* WT in soybean rhizosphere with or without the inoculation of bacteria. The results revealed a significant reduction in the biomass ratio of the *ΔFg12* mutant to that of *F. graminearum* WT under bacterial inoculation compared to buffer‐inoculation conditions (Figure 5F), strongly validating the hypothesis that Fg12 enhances fungal ecological fitness by counteracting bacterial antagonism in the rhizosphere. Thus, these data together imply that soybean rhizosphere *Bacillus* or *Pseudomonas* can effectively prevent *F. graminearum* infection, while *F. graminearum* can secrete Fg12 to effectively inhibit the growth of these bacteria, thereby facilitating niche colonization of *F. graminearum* in the host rhizosphere. Moreover, treatment of surface‐sterilized alfalfa seeds with *B. velezensis*, *P. putida* and syncom also negatively affected *F. oxysporum* root rot disease development (Figure S17A–F, Supporting Information), suggesting that bacteria transplantation between legumes could effectively control *Fusarium* disease.

### 2.6 GMP Efficiently Inhibits Fg12 RNase Activity and Blocks the Inhibition of Fg12 on Soybean‐Associated Bacterial Isolates

**Figure 6:**
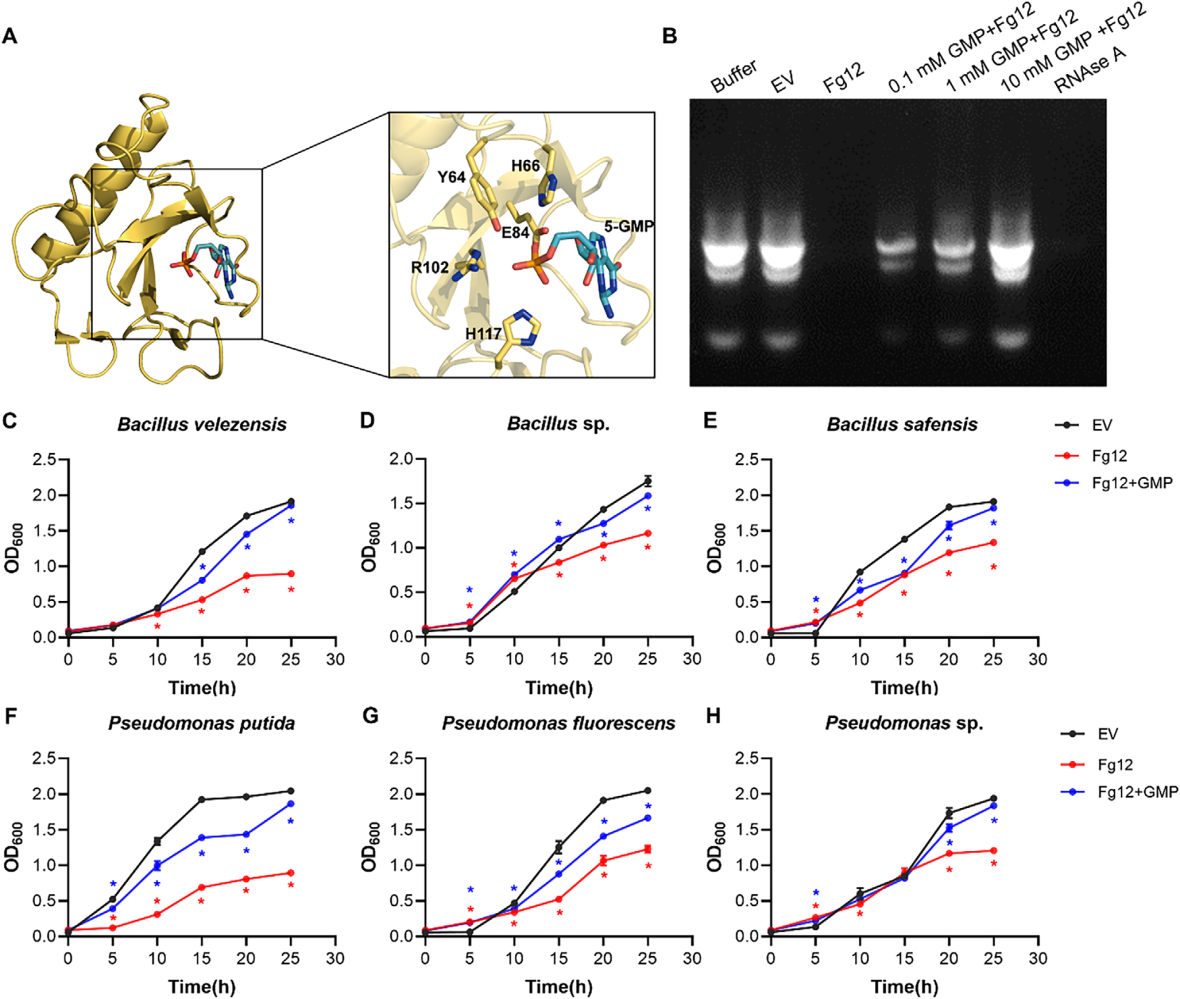
GMP inhibits Fg12 RNase activity and antagonizes Fg12‐mediated inhibition of soybean‐associated bacteria. A) Structural interaction between Fg12 and GMP. Catalytic residues (Y64, H66, E84, R102, H117) and bound GMP are represented as stick models, with residues labeled by single‐letter codes. B) Dose‐dependent inhibition of Fg12 RNase activity by GMP. Recombinant Fg12 (1 µM, expressed in *Escherichia coli*) hydrolyzed soybean root RNA in vitro. RNase A (positive control), buffer, and EV (1 µM of EV protein, negative control) were included. Reactions incubated at 25 °C for 30 min. C‐H) GMP counteracts Fg12‐induced growth suppression of *Bacillus* and *Pseudomonas* in Lysogeny Broth (LB) medium. Bacterial cultures were treated with 10 µM Fg12 (or 10 µM EV control) ± 1 mM GMP. OD_600_ values show mean ± SD of three biological replicates. Asterisks indicate significant differences (unpaired *t*‐test, *p* < 0.05).

Given the aforementioned findings confirming Fg12 as a core effector for *F. graminearum* survival in both soil and host plants, we aim to screen specific compounds targeting Fg12 for disease control. Fg12 is a canonical secreted protein that belongs to the guanyl‐specific fungal RNases family.^[^ ^22^ ^]^ *Aspergillus oryzae* secreted RNase T1 (*Ao*RNase T1, EC 3.1.4.8) is the best investigated member of this group. The crystal structures of AoRNase T1 in complexes with its specific inhibitors, such as 2'‐guanosine monophosphate (2'‐GMP, PDB ID: 1RNT) and 3'‐guanosine monophosphate (3'‐GMP, PDB ID: 1RLS), have been reported previously.^[^ ^26^, ^27^ ^]^ Sequence alignment results showed >60% similarity between Fg12 and *Ao*RNase T1 (data not shown). For structural comparison, the Fg12 protein structure was predicted using AlphaFold (Figure S18A,B, Supporting Information). The overall structure of Fg12 and RNase T1 had high structural similarity, with an RMSD of 0.513 Å for 80 Cα atoms to *Ao*RNase T1 (Figure S18A,B, Supporting Information). Notably, in silico molecular docking analysis revealed conserved binding sites for different isomerides of GMP (2'‐GMP, 3'‐GMP, or 5’‐GMP) and Fg12 (Figure S18A,B, Supporting Information), implying that GMP can effectively inhibit RNase activity. The predicted Fg12–5’‐GMP complex structure revealed that GMP probably binds to Tyr64, His66, Glu84, Arg102 and His117 within the active site region of Fg12 (**Figure** **6**A). To verify whether these conserved residues contribute to the inhibitory effect of GMP on Fg12 RNase activity, we assessed the RNase activity of the purified Fg12 proteins by incubating it with soybean total RNA, GMP solutions with different concentration gradients were added to the reaction system. Indeed, the recombinant Fg12 protein degraded the RNA at the concentration of 1 µM within 30 min of incubation: RNA bands were degraded in samples treated with Fg12, whereas EV protein and protein solution buffer failed to degrade the RNA at the same concentration (Figure 6B). Recombinant RNase A was used as a positive control (Figure 6B). Importantly, when different concentrations of GMP and Fg12 were added at the same time, even 0.1 mM of GMP could inhibit the degradation of RNA by Fg12 compared with no GMP control, and the inhibitory effect increased with the increase of GMP concentration (Figure 6B; Figure S19, Supporting Information). Next, we incubated *B. safensis*, *B. velezensis*, *B. sp*.*, P. putida*, *P. fluorescens* and *P. sp*., with Fg12 or Fg12 together with 1 mM of GMP, and monitored their growth in vitro. *P. polymyxa* was used as a control. The results showed that the addition of 1 mM GMP effectively increased the growth of six tested *Bacillus* and *Pseudomonas* isolates (Figure 6C), but not *P. polymyxa* (Figure S20, Supporting Information). As a control, the addition of 1 mM GMP alone or the addition of Fg12‐5A+GMP had no significant effect on bacterial growth (Figure S21, Supporting Information). These data indicate that GMP can efficiently block the inhibitory activity of Fg12 on soybean‐associated *Bacillus* or *Pseudomonas* isolates.

### 2.7 GMP Alleviates the Symptoms of Fusarium Infection on Soybean

**Figure 7:**
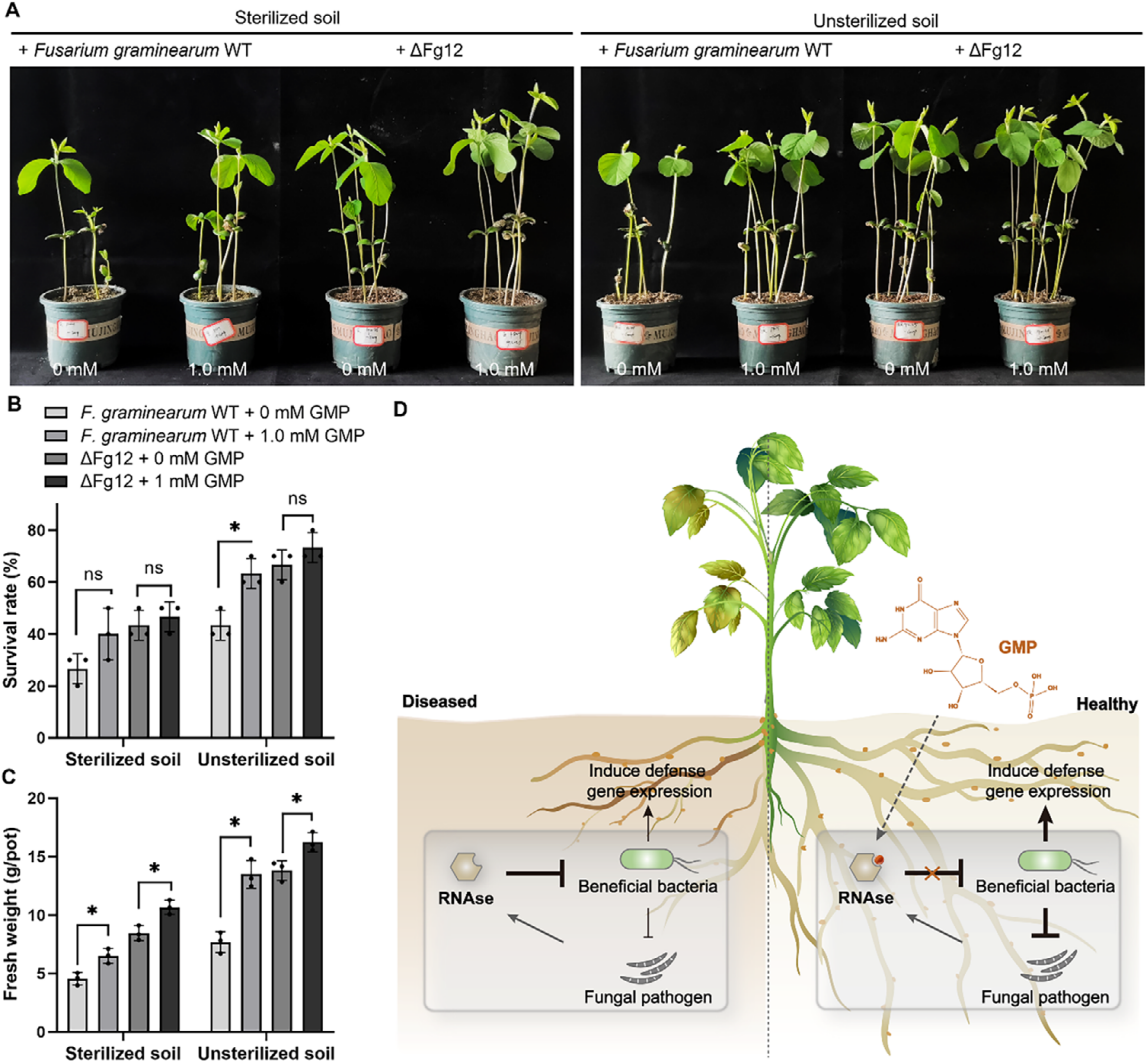
GMP mitigates *Fusarium graminearum* infection symptoms in soybean. A) Phenotypes of soybean plants (cv. Hefeng47) 14 days post‐inoculation with wild‐type *F. graminearum* or ΔFg12. Surface‐sterilized seeds were cultivated in soil (30% sterilized vermiculite) amended with fungal mycelium, followed by application of 10 mL GMP solutions or sterile water. Each pot contained 10 seeds with three independent replicates. B) Survival rates and C) fresh biomass of soybean plants 14 days post‐sowing. Asterisks indicate statistically significant differences (unpaired *t*‐test, *p* < 0.05; N = 3). D) Proposed mechanism: GMP limits fungal infection by inhibiting Fg12 RNase activity, thereby blocking Fg12‐mediated suppression of antifungal bacteria (synthesized from current and previous findings).

Since GMP can effectively inhibit the RNase activity of Fg12, we next investigated whether GMP could be utilized as an anti‐disease compound to manage soybean *Fusarium* root rot. We applied GMP at a concentration of 1 mM during inoculation with *F. graminearum* WT or ΔFg12 mutant under sterilized and non‐sterilized soil conditions. Notably, under sterilized soil conditions, GMP supplementation increased soybean fresh weight by 47.2% (*F. graminearum* WT‐inoculated) and 21.9% (ΔFg12‐inoculated), although no significant improvement in survival rate was observed (**Figure** **7**A–C). In contrast, in non‐sterilized soil, GMP markedly alleviated *Fusarium* disease symptoms in soybean, elevating fresh weight by 75.5% (WT‐inoculated) and 17.5% (ΔFg12‐inoculated) (Figure 7A–C). Furthermore, GMP significantly enhanced the survival rate of WT‐inoculated soybeans by 20%, while no such effect was detected in ΔFg12‐inoculated plants (Figure 7B). Collectively, these results indicate that the reduction of the symptoms of *F. graminearum* infection in soybean by GMP depended on the presence of soil microbes. Additionally, GMP also remarkably increased the survival rate and biomass of soybean upon infection with *F. oxysporum* or *R. solani* (Figure S22A–F, Supporting Information). Similarly, alfalfa plants inoculated with *F. oxysporum* had higher survival rate and biomass in the presence of GMP (Figure S23A–C, Supporting Information). Notably, the biomass of soybean, alfalfa or rice treated with GMP was significantly higher than those of untreated controls (Figure S24A–F, Supporting Information). These results demonstrate that GMP is effective not only in attenuating plant fungal diseases but also in promoting plant growth. To evaluate whether GMP affects the growth of fungal pathogens directly, *F. graminearum* WT, *F. oxysporum* and *R. solani* were cultured on potato dextrose agar (PDA) plates containing different concentrations of GMP. Compared with the control without GMP, there were no significant differences in colony morphology or size were observed at 0.1 mM or 1 mM GMP added (Figure S25A,B, Supporting Information).

## 3 Discussion

Phytopathogens pose a significant threat to plant health. Studies focusing solely on the binary interaction between plants and pathogens often overlook the influence of environmental factors on disease manifestation. Plants cultivated in natural soil are colonized by complex microbial communities, collectively referred to as the microbiota or the plant's second genome, which play a pivotal role in defending against pathogen invasion.^[^ ^31^, ^32^ ^]^ Soil‐borne pathogens must compete with resident soil microbes for ecological niches and overcome the plant microbiota barrier to establish infection. Therefore, it is not surprising that pathogens have evolved mechanisms to counteract antagonistic microbes to promote soil and host colonization. Microbially secreted molecules, including secondary metabolites and effectors, play essential roles in intermicrobial competition.^[^ ^8^, ^9^, ^33^, ^34^ ^]^ In our study, we demonstrated that the *Fg12* gene encoding a secreted RNase in *F. graminearum* is up‐regulated in the soil environment. While the specific soil components inducing its expression remain to be identified, we hypothesize that soil pH, nutrient status, microorganisms, or other environmental factors may contribute significantly to this regulatory process.^[^ ^35^, ^36^, ^37^ ^]^ A microbiome analysis revealed that Fg12 effectively inhibits *Bacillus* and *Pseudomonas*. Both *Bacillus* and *Pseudomonas* are widely recognized as rhizosphere‐beneficial bacteria that protect plants from pathogen infection by directly inhibiting pathogens or inducing plant immunity.^[^ ^11^, ^33^, ^38^, ^39^ ^]^ Our data indicate that several beneficial species of *Bacillus* and *Pseudomonas* effectively suppress soybean root rot when applied to seeds (Figure 5D). Similarly, the soil‐borne pathogen *F. oxysporum*, *V. dahliae* and *Rosellinia necatrix* employ antimicrobial effectors to selectively manipulate host microbiota.^[^ ^8^, ^10^, ^34^, ^40^ ^]^ The obligate parasitic protist *Albugo candida* selectively inhibits phyllosphere‐associated bacteria through antibacterial effectors under apoplastic conditions.^[^ ^41^ ^]^ The human pathogen *Vibrio cholerae*, the etiological agent of cholera, uses a type VI secretion system to deliver antibacterial effectors targeting host‐associated microbiota and facilitate its colonization of the gut.^[^ ^42^ ^]^ Collectively, these findings suggest that effector‐mediated manipulation of host microbiota is a common strategy evolved by a wide variety of pathogens in different habitats. Notably, this paradigm is further complicated by context‐dependent microbial interactions. When soybean plants were inoculated with identical bacterial strains or syncom, no significant difference in fresh weight increment was observed between the *F. graminearum* WT and ΔFg12 mutant treatment groups (Figure 5D,E). This discrepancy may arise from exogenous bacterial loads exceeding baseline soil levels, potentially diluting the functional effect of Fg12. These findings highlight those tripartite interactions among plants, pathogens, and soil microbiomes involve multifaceted complexities that extend beyond our current understanding of effector‐mediated manipulation alone.

Our results show that Fg12 selectively inhibits *Bacillus* and *Pseudomonas*. Notably, certain effectors from distinct pathogens exhibit microbe‐specific antagonism. For example, the *V. dahliae*‐secreted VdAve1 suppresses the growth of Sphingomonadales,^[^ ^8^ ^]^ and the secretion of Fo18438 by *F. oxysporum* reduces the abundance of *Burkholderia*.^[^ ^34^ ^]^ We speculate that the specificity of this inhibition may arise from several factors: first, the target of the effector is conserved in the inhibited bacteria, but not present or mutated in other bacteria; second, some bacteria may produce enzymes or inhibitors that degrade or inhibit the function of the effector. Another possibility is that plants can specifically enrich beneficial microbes to antagonize pathogens under particular environmental conditions, while different pathogens have evolved specific mechanisms against particular antagonistic microbes to maintain their ecological niche and promote infection. For instance, plants can recruit beneficial microorganisms such as *Bacillus*, *Pseudomonas*, or other microbes to combat *Fusarium* infections.^[^ ^38^, ^43^, ^44^ ^]^ These beneficial microbes protect plants through multiple mechanisms, including niche competition, secretion of antimicrobial compounds, and induction of host disease resistance.^[^ ^11^, ^44^ ^]^ In response, *Fusarium* employs weapons such as fusaric acid and β‐lactamase to directly or indirectly interfere with the formation of plant‐beneficial microbial alliances.^[^ ^34^, ^45^ ^]^ Therefore, identifying the members of these microbiota that pathogen effectors target is crucial for developing disease‐protective microbial communities and precisely designing syncoms.

Importantly, using a syncom consisting of three strains of *Bacillus* and three strains of *Pseudomonas* significantly reduced the incidence of *F. graminearum* on soybean and *F. oxysporum* on alfalfa, suggesting good compatibility between different strains and the effectiveness of syncom on closely related plants. Indeed, studies have reported that *B. velezensis* can stimulate resident rhizosphere *P. stutzeri* for plant health,^[^ ^46^ ^]^ and *B. licheniformis* and *P. fluorescens* interact positively in promoting plant growth,^[^ ^47^ ^]^ indicating the synergistic effects between *Bacillus* and *Pseudomonas*.^[^ ^48^ ^]^ However, numerous studies indicate that *Bacillus* and *Pseudomonas* often exhibit competitive interactions, driven by secondary metabolites and effectors secreted through the type VI secretion system.^[^ ^11^, ^49^, ^50^ ^]^ For example, mutating the pyoluteorin synthesis genes in *P. protegens* enhances its coexistence with *B. velezensis* and improves the disease control efficacy of their syncom.^[^ ^50^ ^]^ Notably, in Figure 5E, the *P. putida* treatment exhibited significantly lower values compared to the syncom, suggesting potential competitive interactions between the *Pseudomonas* and *Bacillus* strains within the syncom. Therefore, the construction of syncom in the current study requires further optimization. Additionally, the observed biocontrol effects of individual bacteria or syncom on plant diseases were evaluated only under pot conditions, necessitating further validation in field trials. Third, although our study has revealed the antimicrobial activity of Fg12, the precise mechanisms underlying its antibacterial action and selective inhibition against distinct bacterial species remain unclear, requiring further investigations through approaches such as bacterial mutagenesis screening and protein interaction studies.

Increasing evidence shows that pathogen virulence effectors can be potential targets for developing anti‐disease agents, and solving the 3D models of these effectors is crucial for screening and designing specific chemical blockers.^[^ ^15^, ^16^, ^17^ ^]^ Notably, AvrE‐family effectors, which function as water‐ and solute‐permeable channels, are major virulence factors responsible for bacterial multiplication and infection. Targeted screening of blockers based on the structure of DspE identified polyamidoamine dendrimers as inhibitors of the DspE/AvrE channels, which broadly inhibit AvrE virulence activity and reduce bacterial infection.^[^ ^17^ ^]^ FY21001, a compound designed to target the virulence effector of *Magnaporthe oryzae*, can effectively inhibit its function and reduce the occurrence of rice blast.^[^ ^15^ ^]^ Our previous study showed that Fg12 is an important virulence factor of *F. graminearum*. Fg12 targets plant to induce plant cell death, and mutation of the Fg12 gene impaired the pathogenicity of *F. graminearum*.^[^ ^22^ ^]^ In the present study, we show that Fg12 also plays a crucial role in soil colonization, and its antimicrobial activity in modulating soil microbiota partially contributes to fungal virulence. These results suggest that Fg12 is essential for both surviving in soil and infecting plants. Thus, we speculate that it could be a potential antifungal target. Fg12 is a fungal‐secreted guanyl‐specific RNase. Through structural simulation, molecular docking, and functional verification, we found that its inhibitor, GMP, can effectively inhibit the RNase activity of Fg12. When treating plants with GMP, it effectively prevented and controlled root rot in both soybean and alfalfa. By inhibiting Fg12 enzymatic activity, GMP blocks both its microbiome‐disrupting and plant‐pathogenic functions (Figure 7D). Importantly, Fg12 is widely distributed among various fungal pathogens, suggesting that GMP may serve as a broad‐spectrum agent targeting diverse pathogenic fungi. Indeed, we found that GMP is also effective in preventing root rot in legumes caused by *F. oxysporum* and *Rhizoctonia solani*. Although Fg12‐type RNases are conserved across diverse fungal species, their functions are not always consistent with Fg12. For example, the secreted RNase SRE1 contributes to *S. turcica* virulence,^[^ ^24^ ^]^ while Ribo1, a secreted RNase of *Ustilago maydis*, is dispensable for the virulence.^[^ ^51^ ^]^ Therefore, the effect of GMP on preventing and controlling different pathogens needs to be further determined. More interestingly, many RNase‐like effectors in *Blumeria graminis*, termed RALPH, have structural similarity to RNase T1 and are required for full pathogenic development.^[^ ^52^, ^53^ ^]^ However, RALPH effectors lack RNase activity. Whether GMP can bind to them and interfere with their virulence functions remains to be further investigated.

In summary, our study reveals that *F. graminearum* secretes the virulence factor Fg12 to promote its own colonization in soil and facilitate infection of host plants. As an RNase, Fg12 inhibits *Bacillus* and *Pseudomonas* species in the soybean rhizosphere microbiome, which protect plants by antagonizing *F. graminearum* and inducing defense gene expression. The exogenous application of GMP, a compound that specifically inhibits the RNase enzymatic activity of Fg12, effectively reduces soybean root rot caused by *F. graminearum* (Figure 7D).

## 4 Experimental Section

### Bacterial, Fungal and Plant Growth Conditions

Soybean (*Glycine max* Merrill), alfalfa (*Medicago sativa*), rice (*Oryza sativa*) plants were cultivated in a glasshouse at 22–25 °C with a 16‐h light/8‐h dark cycle. *Agrobacterium tumefaciens *GV3101, *Escherichia coli *BL21 and JM109 strains were cultured on Luria‐Bertani (LB) medium (BeyoPure, Shanghai) with appropriate antibiotics at 28or 37 °C. *Fusarium graminearum* PH‐1, *F. oxysporum*, *Rhizoctonia solani, B. subtilis* strain OKB105 and *Phytophthora sojae* P6497 were stored in our laboratory. The fungi were cultured on Potato Dextrose Agar (PDA) medium (Hopebio, Qingdao), whereas *P. sojae* was cultured on V8 medium (Rayzbio, Shanghai). Various bacteria (*Bacillus sp*., *B. safensis*, *B. velezensis*, *Pseudomonas sp*., *P. putida*, *P. fluorescens*, and *Paenibacillus polymyxa*) were isolated from soybean rhizosphere soil and roots. The complete protocol for microbial isolation and identification is provided in the following section.

### Bacterial Isolation and Identification

Soybean roots were carefully excavated, and loosely attached soil was manually shaken off. The rhizosphere soil was homogenized using a tissue lyser with 3 mm metal beads at 5000 rpm for 30 s, followed by serial dilution in sterilized water. Subsequently, 100 µL of the diluted rhizosphere soil samples were plated on bacterial growth media and incubated at 28 °C for 5 days. Soybean roots were cut into 2–4 cm segments, homogenized using a tissue lyser with 3 mm metal beads at 5000 rpm for 30 s, and then subjected to isolation on bacterial growth media. Single colonies were randomly selected, and PCR coupled with Sanger sequencing using 27F and 534R primers targeting the 16S rRNA gene was performed. Taxonomic classification of the isolates was determined by comparing the sequencing data with the SILVA 138 database.

### Soil Collection, Soil Extract Preparation and Fg12 Gene Expression Analysis

Soil for experiments was collected from a soybean field in the Lishui district, Nanjing, China (Baima teaching and research base). The soil was mixed with 30% sterile vermiculite to improve handling. Soil extract was prepared by mixing 150 g of dry soil with 500 mL of distilled water. Agitating soil slurry on a rotary shaker for 20 min at 28 °C at 180 rpm, and subsequently autoclaved for 15 min at 121 °C. Soil precipitate was collected by centrifugation at 8000 g for 10 min at 4 °C and the supernatant was collected and stored at −20 °C until use. The soil extract solution was mixed with Potato Dextrose Broth (PDB) medium in different proportions (v:v), and the final concentration of the soil extract solution was 25%, 50%, and 75%, respectively. *F. graminearum* was cultured using different concentrations of the soil extract, followed by RNA extraction and real‐time PCR analysis for Fg12 gene expression relative to *Fg‐tubulin*. Primer pairs used in this experiment are shown in Table S8, Supporting Information.

### Root Microbiome Analysis

Mycelial agar plug inoculation method was used for pathogen inoculation.^[^ ^54^ ^]^ *F. graminearum* isolate PH‐1 or Fg12 deletion mutant was grown in 9 cm Petri dishes (Hailun Biomedical company, Nantong) containing 15 mL PDA medium and cultured for 7 days at 25 °C. After the mycelium completely covered the plate, the mycelium was chopped together with the medium and mixed with soil. Three dishes of medium were added to each pot (The diameter of the pot is 13 cm and the height is 13 cm, Mujinghao, Nanjing). Hefeng47 soybean seeds were surface‐sterilized and grown in soil (with 30% sterilized vermiculite) mixed with *F. graminearum* or Fg12 deletion mutant mycelium pieces, with blank PDA medium used as a non‐inoculation control. Each pot contained 10 soybean seeds, and the experiment was independently repeated three times. Roots of each soybean plant were dug out, and loosely attached soil was removed by manual shaking, while rhizosphere soil was collected from the root surface. Rhizosphere soil from the roots of three soybean plants was pooled to form a single biological replicate. Six samples were collected from each treatment group, and five of the six samples were randomly selected for further analysis. Samples were flash‐frozen in liquid nitrogen, and genomic DNA was isolated using a Power Soil DNA Isolation kit (MoBio Laboratories, Inc.) according to the manufacturer's instructions. DNA quality was checked on a 1.0% agarose gel and a NanoDrop2000 spectrophotometer (Thermo Scientific, USA) and stored at −80 °C. The hypervariable region V3‐V4 of the bacterial 16S rRNA gene and the fungal ITS gene were amplified with primers 338F/806R and ITS1F/ITS2R (Table S8, Supporting Information) using an ABI GeneAmp 9700 PCR thermocycler (Applied Biosystems, USA), respectively. PCR conditions were as follows: initial denaturation at 95 °C for 3 min, followed by 27 cycles for 16S and 35 cycles for ITS of 30 s at 95 °C, 30 s at 55 °C, 30 s at 72 °C, and a final elongation at 72 °C for 10 min. Purified amplicons were pooled in equimolar amounts and sequenced using an Illumina MiSeq PE300 platform (Illumina, San Diego, USA) following standard protocols by Majorbio Bio‐Pharm Technology Co. Ltd. (Shanghai, China). Raw sequencing data were processed using the QIIME (Quantitative Insights into Microbial Ecology) software package.^[^ ^55^ ^]^ Low‐quality sequences, including those shorter than 150 bp or containing mononucleotide repeats, were removed, and filtered paired‐end reads were assembled using FLASH software.^[^ ^56^ ^]^ Bacterial sequences with a 97% similarity threshold were clustered into operational taxonomic units (OTUs) by comparison with the SILVA (Release 128, http://www.arb‐silva.de) and NCBI (Release 6.0, http://unite.ut.ee/index.php) databases.^[^ ^57^, ^58^ ^]^ Microbial richness and the Shannon index were calculated using OTU data with the ‘vegan’ package in R (version 3.5.1), and functional profiles of bacterial communities were annotated using FAPROTAX databases.^[^ ^59^ ^]^ Principal coordinates analyses (PCoAs) of Bray‐Curtis distances were performed using the “pcoa” function in the R package.

### Soil Colonization Assays

Soil was placed into sterilization bags (305 × 430 mm, Tianrun, Anhui, China) that are permissive to autoclaving and a soil depth of 2–3 cm. Each sample was autoclaved for 30 min at 121 °C and 18 psi, and bring to cooled to room temperature immediately after autoclaving. Then autoclave (30 min at 121 °C, 18 psi) the soil a second time. Unsterilized or sterilized soil (with 30% sterilized vermiculite) was mixed with *F. graminearum* or Fg12 deletion mutant mycelium pieces and incubated in a glasshouse at 25 °C with a 16‐h light/8‐h dark cycle for 7 days. Genomic DNA from different treatments was isolated using a Power Soil DNA Isolation kit (MoBio Laboratories, Inc.) according to the manufacturer's instructions. *F. graminearum* biomass was determined by real‐time PCR using *F. graminearum*‐specific primers (FgITS‐F/R) targeting the ITS region of the ribosomal DNA. Primers (16s‐340F/533R) targeting a conserved region of the bacterial 16S rRNA gene were used for normalization. These primers are listed in Table S8, Supporting Information. SYBR qPCR Master Mix (Vazyme) and an CFX96 Real‐Time PCR Detection System (BioRad Laboratories) were used for this analysis. Real‐time PCR conditions were as follows: an initial denaturation at 95 °C for 30 s, followed by 40 cycles for 5 s at 95 °C, for 34 s at 60 °C, and dissociation at 95 °C for 15 s, 60 °C for 60 s, and 95 °C for 15 s.

### Plasmid Construction

The *Fg12* gene encoding the mature protein and the Fg12 mutant (Fg12‐5A, loss of RNase activity) were cloned using the cDNA of *F. graminearum* PH‐1 as a template.^[^ ^22^ ^]^ PCR amplification was performed with high‐fidelity enzyme 2×Phanta Max Master Mix (Vazyme, China). The amplified fragments were ligated into the pFlag‐His vector using the In‐Fusion Cloning Kit (Vazyme Biotech, Nanjing, China). Individual colonies of each construct were screened by PCR and verified by sequencing.

### Production and Purification of Recombinant Fg12 and Fg12‐5A Proteins

pFlag‐Fg12‐His, pFlag‐Fg12‐5A‐His, or empty vector pFlag‐GST‐His was transformed into *E. coli* strain BL‐21, and positive transformants were cultured overnight at 37 °C in LB medium with constant shaking at 200 rpm. Subsequently, cells were diluted (1:50) in LB medium and grown at 37 °C to an optical density (OD_600_) of 0.6. Heterologous protein expression was induced with 0.4 mM isopropyl β‐D‐thiogalactoside (IPTG) for 12 h at 18 °C. The supernatant was centrifuged and filter sterilized to eliminate *E. coli* cells. Proteins were precipitated overnight at 4 °C by adding 70 g ammonium sulphate per 100 mL supernatant. The precipitate was collected by centrifugation at 12 000g for 10 min at 4 °C and resuspended in 10 mM Tris‐HCl (pH 7.5) and 1 mM EDTA. Recombinant proteins were purified using the AKTA Avant 25 system (GE Healthcare) with a HisSep Ni‐NTA 6FF Chromatography Column (5 mL, 20504ES08; Yeasen Biotech) and HiTrap 26/10 Desalting Prepacked Columns (10262891; GE Healthcare). The proteins were concentrated using Millipore ultrafiltration tubes (Amicon Ultra, Ultracel‐10K), then dispensed into 2 mL EP tubes and stored at −70 °C. Protein concentration was determined using a Bradford Protein Assay Kit (Beyotime Biotechnology). pBE‐S‐Fg12‐His, pBE‐S‐Fg12‐5A‐His or pBE‐S empty vector (Takara) was transformed into *B. subtilis* strain OKB105. *B. subtilis* was transformed according to the method described previously.^[^ ^30^, ^60^ ^]^

### Generation of *F. graminearum* Fg12‐Deletion Mutant

The *F. graminearum* Fg12‐deletion mutant (ΔFg12) was constructed previously using a polyethylene glycol‐mediated protoplast transformation method.^[^ ^22^ ^]^ Transformants were screened by PCR. ΔFg12 mutants were cultured on PDA plates at 25 °C in the dark.

### Detection of Inducible Resistance of Bacteria to Soybean Root

The induced resistance of bacteria to soybean was detected according to the method revised from Xia et al.^[^ ^44^ ^]^ Etiolated soybean seedlings were collected 4 days after sowing. The roots were kept in the bacterial solutions for 12 h in the dark. The control group was treated with sterile water. The roots were collected for RNA extraction and gene expression analysis.

### In Vitro Microbial Growth Inhibition Assays

Bacterial isolates were grown on LB medium at 28 °C. Single colonies of each bacterium were selected and grown at 28 °C and 220 rpm for 12 h. Bacterial solutions were resuspended to OD_600_ = 0.05 in LB medium supplemented with purified Fg12 or Fg12‐5A protein (10µM). For microbial antibiotic sensitivity test, bacterial suspension (200 µL, OD_600_ = 0.05) was spread on a 90 mm diameter LB agar plate and allowed to dry. A 5 mm diameter filter paper disc was placed on the dried LB agar plate, and 20 µL of 10 µM protein solution was added to the disc. The plates were incubated at 28 °C for 12 h. For fungal and oomycete spore collection, spores were harvested from PDA and V8 agar plates, diluted, and mixed with protein solution (10µM) in liquid PDB or V8 culture medium. The final spore concentration was adjusted to 10^4^ spores per milliliter. Subsequently, 200 µL of the microbial suspension was added to each well of a transparent 96‐well flat‐bottom polystyrene tissue culture plate. The plate was then placed in a Tecan microplate reader (Infinite 200 PRO) to monitor microbial growth. Bacterial growth was measured at 28 °C, while fungal growth was measured at 25 °C. The plate was shaken every 2 h with a dual‐axis oscillation at 200 rpm for 10 s. OD_600_ values of each bacterial suspension were measured every 5 h, while OD_600_ values of each fungal or oomycete suspension were measured every 12 h. Each treatment was replicated three times. To detect the effect of GMP on the bacteriostatic function of Fg12, GMP solution was added to the bacterial suspension reaction system with Fg12 or Fg12‐5A protein. To detect the influence of GMP on *F. graminearum* growth, *F. graminearum* PDA disks were placed in the center of petri dishes containing 15 mL of PDA medium with different concentrations of GMP. Three replicates were conducted. Pictures were taken after 4 days of incubation at 25 °C.

### Microbial Confrontation Assays

Bacterial isolates were grown on LB medium at 28 °C. Single colonies of each bacterium were selected and grown at 28 °C and 220 rpm for 12 h. Bacterial solutions were resuspended to OD_600_ = 0.1 with 10 mM MgSO_4_. Fifteen mL of PDA medium was added to each petri dish (90 mm diameter). *F. graminearum* PDA disks were placed in the center of petri dishes, and sterilized filter paper soaked in individual bacterial solutions was placed around the fungus. Sterilized filter paper soaked in 10 mM MgSO_4_ buffer was used as controls. Three replicates were conducted. Pictures were taken after 4 days of incubation at 25 °C.

### Pathogen Inoculation under Potted Conditions

Hefeng47 soybean seeds were surface‐sterilized and grown in soil (with 30% sterilized vermiculite) mixed with *F. graminearum* or Fg12 deletion mutant mycelium pieces, with blank PDA medium used as a non‐inoculation control. Each pot contained 10 soybean seeds, and the experiment was independently repeated three times. For individual bacterial inoculations, overnight cultures of different *Bacillus* and *Pseudomonas* isolates were resuspended with 10 mM MgSO_4_ buffer and diluted to a final OD_600_ of 0.1. For Syncom inoculation, overnight cultures of six individual bacterial strains were resuspended in 10 mM MgSO_4_ buffer and adjusted to the same OD_600_. Equal volumes of each bacterial suspension were pooled, and the OD_600_ of the suspensions was adjusted to a final OD_600_ = 0.1. Then, 10 mL of the inoculum or 10 mM MgSO_4_ buffer was added to each pot. After 14 days post‐inoculation, the survival rate and fresh weight of soybean were recorded. Each treatment was replicated three times.

### Preparation and Purification of Proteins Secreted from *F. graminearum* WT and ΔFg12

The preparation method of soil extract and 25% PDB medium were as described in the materials and methods above. *F. graminearum* WT and ΔFg12 mutant strains were subsequently cultured in 150 mL of 100% and 25% PDB medium, respectively, under conditions of 25 °C and 120 rpm min^−1^ for 7 days. After cultivation, the fungal suspension was centrifuged, and the supernatant was filtered through a 0.22µm bacterial filter. The filtered supernatant was concentrated to 20 mL using a Millipore ultrafiltration tubes (Amicon Ultra, Ultracel‐3K) and then lyophilized into powder using a freeze dryer. Lyophilization parameters: −30 °C under 0.1 Pa vacuum for 48 h.

### Detection of RNase Activity using RNase Activity Fluorescence Assay Kit

The RNase activity of proteins, secreted proteins of *F. graminearum*, and rhizosphere soil was detected using the RNase Activity Fluorescence Assay Kit (P0347S) from Beyotime Biotechnology Co., Ltd (Shanghai). The test samples were diluted to a fixed concentration with 1X Reaction Buffer and set aside. The reaction system for enzyme activity testing consisted of 80 µL 1X Reaction Buffer, 10 µL sample, and 10 µL RNase Substrate, totaling 100 µL. After preparing the reaction system, the mixture was vortexed for 1–2 min to ensure thorough mixing. Fluorescence measurements were immediately conducted using a fluorescence microplate reader. The microplate reader was set to 37 °C, with excitation at 490 nm and emission at 520 nm. Readings were taken every 3 min for a total duration of 30 min, with three replicates per treatment.

### Analysis of the Effects of GMP on Plant Growth and Disease Occurrence

To analyze the effects of GMP on plant growth, soybean (Hefeng47), alfalfa (Zhongmu No.1), and rice (Nipponbare) seeds were surface‐sterilized and grown in solutions with different concentrations of GMP. Fresh weight of each treatment was measured after 14 days of growth in a glasshouse at 22–25 °C with a 16‐h light/8‐h dark cycle. The experiment was independently repeated three times.

To analyze the effects of GMP on plant disease occurrence, soybean seeds were surface‐sterilized and grown in soil (with 30% sterilized vermiculite) mixed with *F. oxysporum* or *R. solani* mycelium pieces, then 10 mL of different concentrations of GMP solutions were poured into different pots. Each pot contained 10 soybean seeds, and the experiment was independently repeated three times. Survival rate and fresh weight of soybean plants from each pot were measured 14 days post‐sowing. The method of *F. oxysporum* inoculation on alfalfa was similar to that of soybean inoculation.

### Molecular Docking of Fg12 and 5’‐GMP

Molecular docking of Fg12 and 5’‐GMP was conducted using the Molecular Operating Environment (MOE) docking suite. AlphaFold was used to predict the tertiary structure of Fg12 proteins. The 2D coordinates of 5’‐GMP were prepared by ChemDraw and converted into PDB format using Phenix software. Ligand‐receptor interactions were docked and analyzed by the “Triangle Matcher” placement method in MOE.

### Phylogenomic Analyses

The full‐length sequence of Fg12 protein was used as BLASTP query to retrieve homologs from other species on NCBI databases. BLAST searches were performed with an E‐value threshold of 1.0E‐5. When multiple homologs from a different strain of the same species were present, one homolog was arbitrarily chosen and retained (with E‐values ranging from 1.4E‐51 to 3.9E‐6). Multiple sequence alignment and maximum likelihood (ML) tree estimation were performed. Sequence alignment was conducted in MAFFT^[^ ^61^ ^]^ and HAlign^[^ ^62^ ^]^ with default settings. TrimAl was used to trim the alignment to eliminate poorly aligned regions.^[^ ^63^ ^]^ The species tree was estimated in RAxML using the standard algorithm.^[^ ^64^ ^]^ Visualization of the tree was performed with iTOL.^[^ ^65^ ^]^ SignalP‐6.0 (https://services.healthtech.dtu.dk/services/SignalP‐6.0/) and EffectorP (https://effectorp.csiro.au/) website were used for the prediction of effector and signal peptide, respectively.^[^ ^66^, ^67^, ^68^ ^]^

### Statistical Analysis

Statistical analysis was conducted using the Statistical Package for the Social Sciences (SPSS 17.0) and R software (Version 4.1.2; Vienna, Austria). Significant differences in microbial variables between two groups were analyzed by the Wilcoxon rank‐sum test with false discovery rate (FDR) adjusted *P* values using the ‘ggpubr’ package. Differences in microbial compositions among groups were determined by permutational multivariate analysis of variance (PERMANOVA) based on Bray‐Curtis distances using the ‘vegan’ package. Dissimilarity matrices were visualized using unconstrained principal coordinates analysis (PCoA) with the ‘vegan’ package. To identify microbial taxa with significantly different relative abundances between groups, differential abundance analysis was performed using STAMP software v2.1.3. Other plots were visualized with the ‘*ggplot2*’ package.

## Conflict of Interest

The authors declare no conflict of interest.

## Author Contributions

B.Y. and S.Y. contributed equally to this work. Y.C.W., B.Y., and S.Y. designed the experiments. B.Y., S.Y., Y.Z., M.H.T., J.Y.Z., W.X.Z., and X.M.W. carried out the experiments. S.Y., X.M.W., Y.W.Z., Y.S.W., W.W.Y., Y.W., K.X.D., and B.Y. analyzed the data. B.Y., X.M.W., Y.C.W., Z.F.G., and S.Y. wrote the manuscript. All authors approved the manuscript.

## Supporting information



Supporting Information

Supporting Information

Supporting Information

Supporting Information

Supporting Information

Supporting Information

Supporting Information

Supporting Information

Supporting Information

Supporting Information

## Data Availability

The raw data were deposited in the National Center for Biotechnology Information (NCBI) Sequence Read Archive (SRA) under accession number PRJNA1127493. Source Data are provided with this paper. The data that support the findings of this study are available from the corresponding author upon reasonable request.
