# Supplementary material for: Inhibition of RNase to Attenuate Fungal‐Manipulated Rhizosphere Microbiome and Diseases
Source: Adv Sci (Weinh). 2025 Aug 7;12(40):e03146. doi: 10.1002/advs.202503146 (PMC12561316; doi:10.1002/advs.202503146)
Supplement: Supplementary file 1 — Supporting Information [file ADVS-12-e03146-s009.docx]

**Supporting Information**

**Inhibition of RNase to** **Attenuate Fungal-Manipulated** **Rhizosphere Microbiome and Diseases**

*Bo Yang^1, 8*^, Sen Yang^2, 8^, Xiaomi Wang^3, 4^, Yuanwei Zhang^5^, Yao Zhao^2^, Menghuan Tao^1^, Jinyi Zhu^6^, Wanxin Zhang^2^, Yansu Wang^7^, Kaixuan Duan^2^, Yan Wang^2^, Wenwu Ye^2^, Zhenfei Guo^1*^, Yuanchao Wang^2*^*


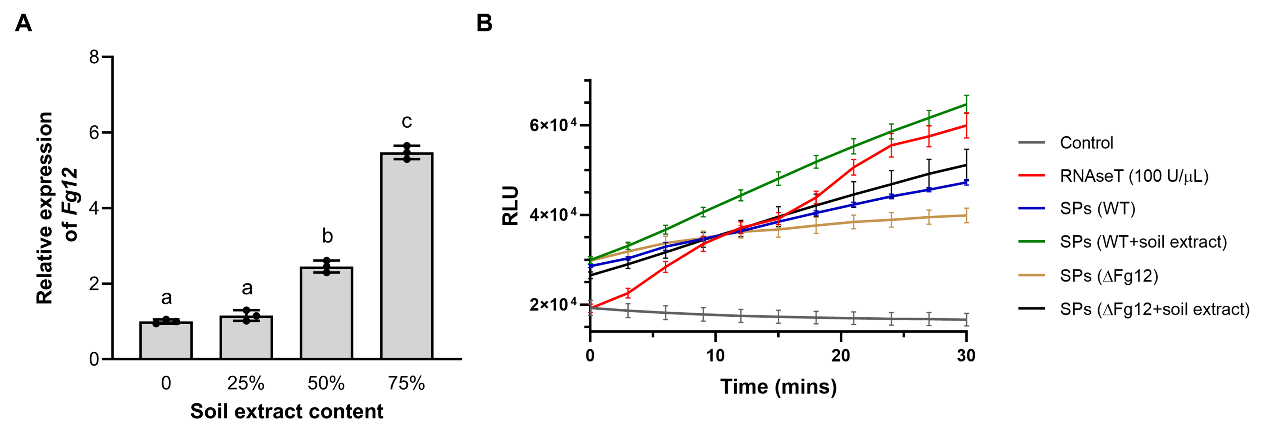


**Figure S1. Fg12 promotes *Fusarium graminearum* soil colonization.**

A) Fg12 expression is upregulated by soil extract supplementation. Expression levels of Fg12 relative to Fg-Tubulin (internal control) in *F. graminearum* cultured for 5 days in pure potato dextrose broth (PDB) or PDB supplemented with increasing soil extract concentrations. Data represent mean ± SD of three biological replicates. Different lowercase letters indicate statistically distinct groups (one-way ANOVA, *P* < 0.05). B) RNase activity of secreted proteins (SPs) from wild-type (WT) *F. graminearum* and ΔFg12 strains cultured in pure PDB or PDB + 75% soil extract. Blank PDB and RNase T served as negative and positive controls, respectively. Activity was measured at 3-min intervals over 30 min. Data represent three biological replicates.


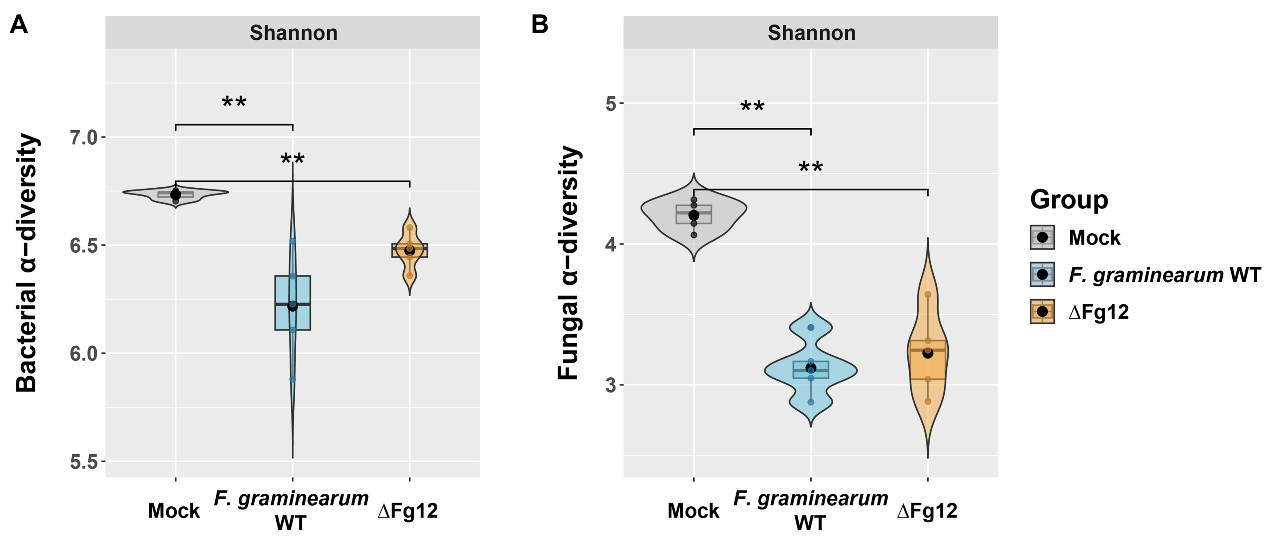


**Figure S2. α-diversity of soybean root microbiomes upon *Fusarium graminearum* infection.** A) *F. graminearum* inoculation changes α-diversity of soybean root bacteriome (wilcox.test, *P* < 0.05, N=5). The plot displays the average Shannon index ± SD. soybean rhizosphere soils. B) *F. graminearum* inoculation changes α-diversity of soybean root mycobiome (wilcox.test, *P* < 0.05, N=5).


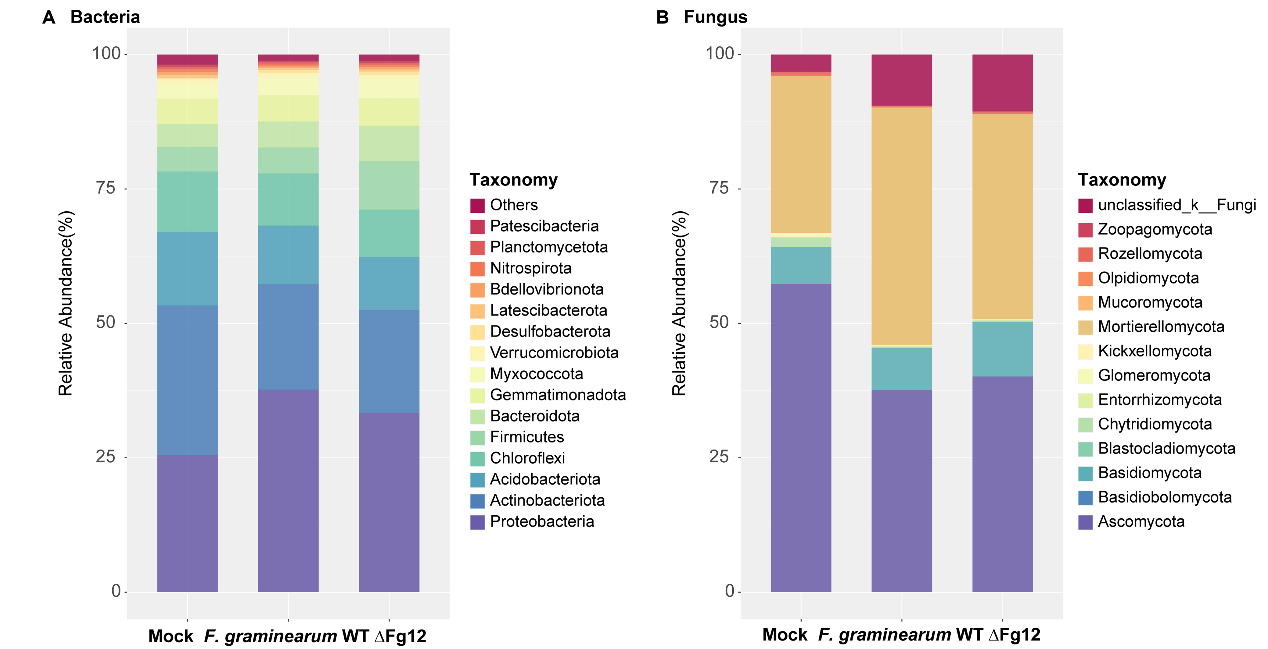


**Figure S3. Taxonomic profile of soybean root microbiomes upon *Fusarium graminearum* infection at the phylum level.** A) Taxonomic profile of bacterial phyla in the root bacteriome of soybean plants 14 days after inoculation with wild-type *F. graminearum* and a *Fg12* deletion mutant as determined by 16S ribosomal DNA profiling. B) Taxonomic profile of fungal phyla in the root mycobiome of soybean plants 14 days after inoculation with wild-type *F. graminearum* and a Fg12 deletion mutant as determined by ITS amplicon sequencing.


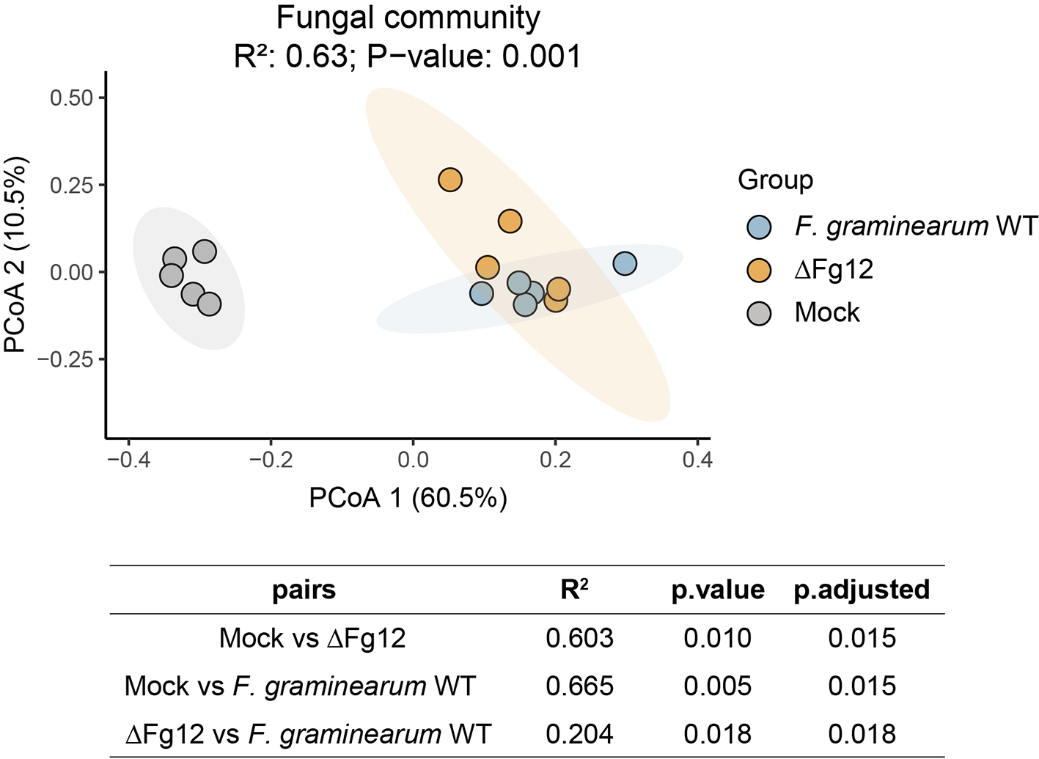


**Figure S4. Fungal community of soybean root microbiomes upon *Fusarium graminearum* infection.** Principal coordinate analysis based on Bray–Curtis dissimilarities reveals separation of root mycobiome compositions 14 days after inoculation with wild-type *F. graminearum* and a Fg12 deletion mutant (PERMANOVA, *P* < 0.01, N = 5). Pair-wise PERMANOVA results are displayed below each PCoA. Roots with rhizosphere soil from three soybean plants were pooled to form a single biological replicate.


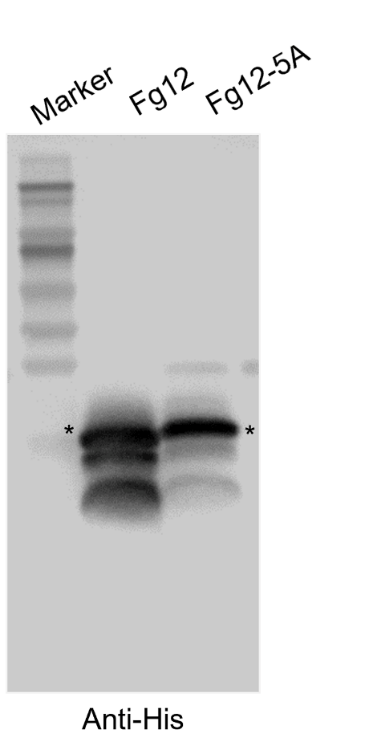


**Figure S5. Detection of Fg12 and Fg12-5A protein secreted by *Escherichia coli*.** Representative immunoblots showing the Fg12 and Fg12-5A protein heterologously produced from *E. coli*. The band representing Fg12 and Fg12-5A are indicated with an asterisk.


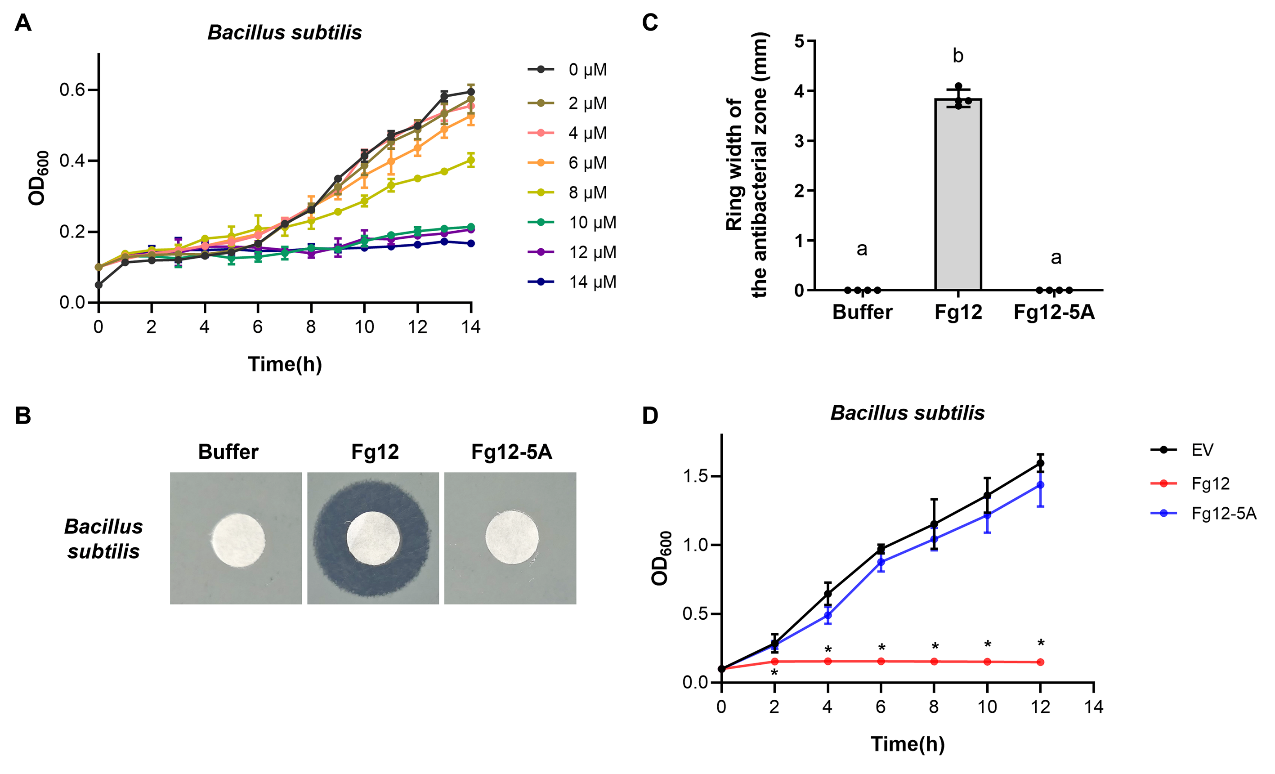


**Figure S6. Fg12 inhibits *Bacillus subtilis* growth in vitro.** A) Minimum inhibitory concentration (MIC) of Fg12 against *B. subtilis* in LB medium. OD_600_ was monitored at 2-h intervals. Data represent mean ± SD (unpaired t-test, N = 3). B) Disk diffusion assay of *B. subtilis* treated with Fg12 (10µM), buffer, or Fg12-5A (10µM). Bacterial plates were prepared with OD_600_ = 0.05 suspensions. Image captured 16 h post-inoculation. C) Antibacterial zone width measured 16 h post-inoculation. Data are mean ± SD of four replicates. Different lowercase letters indicate significant differences (one-way ANOVA, *P* < 0.05). D) Growth curves of IPTG-induced *B. subtilis* OKB105 expressing Fg12 or Fg12-5A in LB medium at 20°C. OD_600_ recorded every 2 h. Data represent mean ± SD (unpaired t-test, N = 3). Asterisks denote significance (*P* < 0.05).


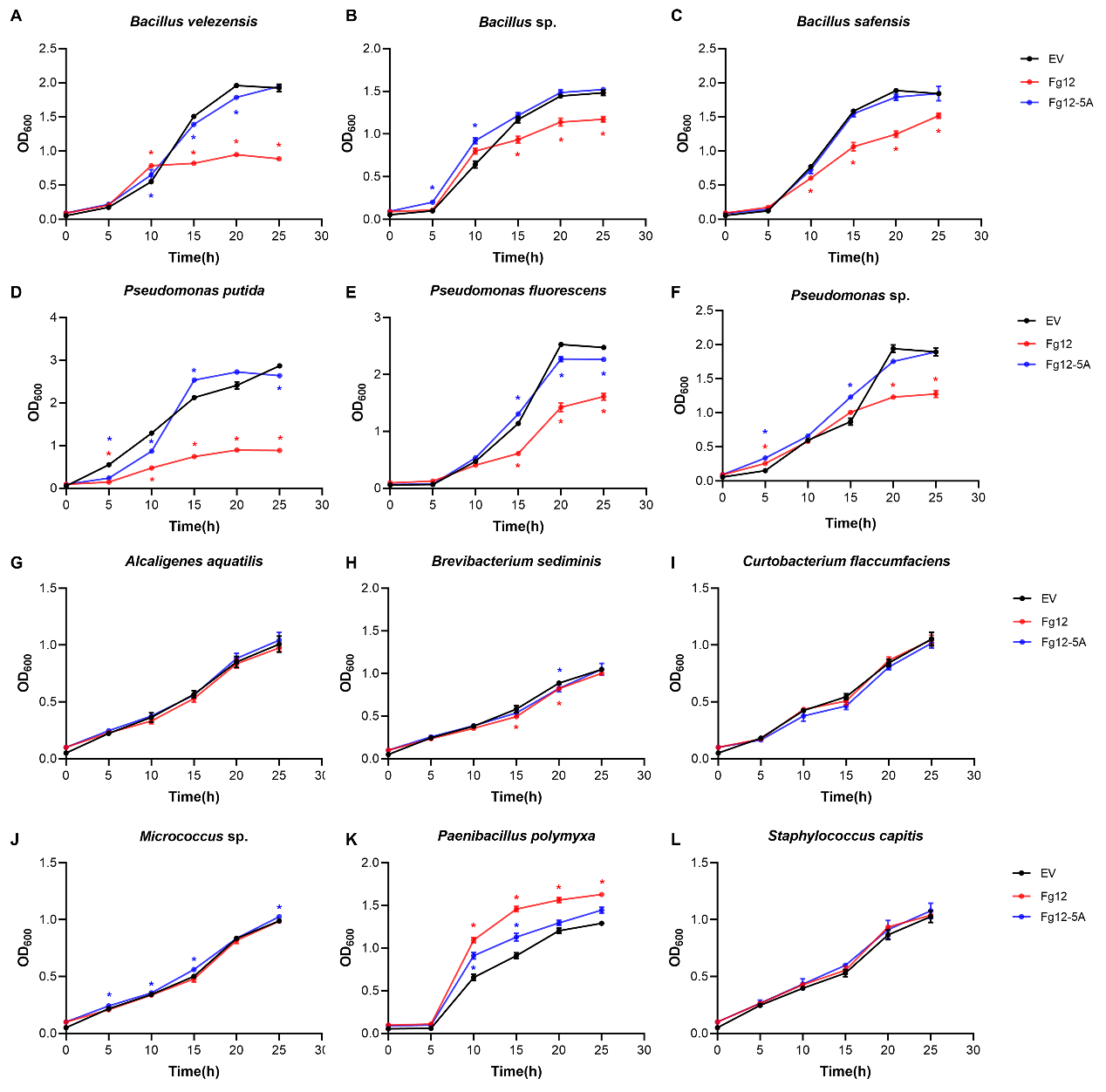


**Figure S7. Fg12 selectively inhibits in vitro growth of soybean-associated bacterial isolates.** A-F) *Bacillus* and *Pseudomonas* are inhibited by Fg12 in lysogeny broth (LB) medium. G-L) *P. polymyxa*, *Alcaligenes aquatilis*, *Curtobacterium flaccumfaciens*, *Micrococcus* sp., *Staphylococcus capitis* and *Brevibacterium sediminis* are not inhibited by Fg12 in LB medium. Recombinant Fg12 protein (10 μM) produced by *E. coli* was used, while EV (glutathione S-transferase, GST protein, 10 μM) and the Fg12 enzyme mutant Fg12-5A protein produced by *E. coli* were used as controls. Graphs display the average OD_600_ of three biological replicates ± SD (unpaired t-test, N = 3). The asterisk represents significant differences (*P* < 0.05).


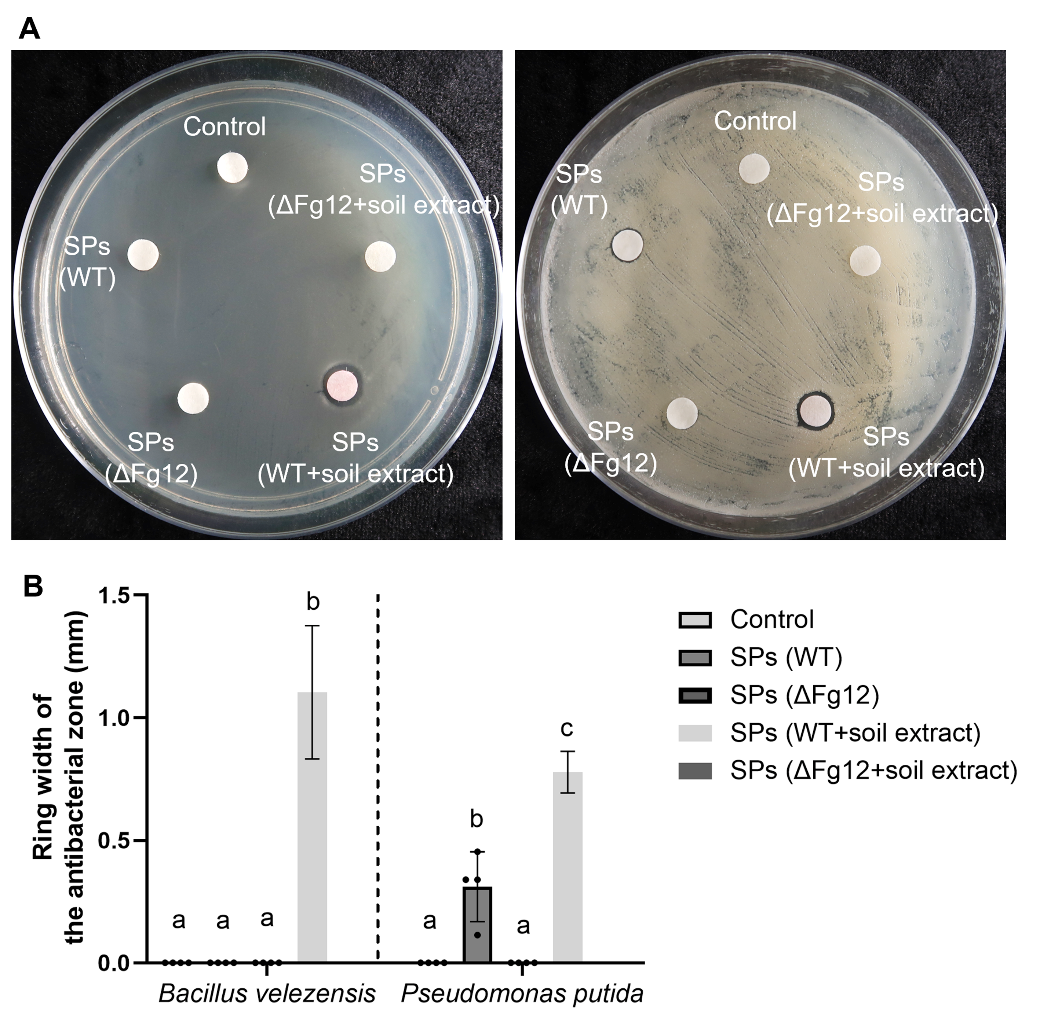


**Figure S8. Antimicrobial activity of *Fusarium graminearum* WT or ΔFg12 secreted proteins against *Bacillus velezensis* and *Pseudomonas putida*.** A) Disk diffusion assay against *B. velezensis* and *P. putida* using secreted proteins from *F. graminearum* WT or ΔFg12. Bacterial plates were prepared with OD_600_ = 0.05 suspensions. Image captured 16 h post-inoculation. B) Antibacterial zone width measured at 16 h. Data represent mean ± SD (N = 4). Different lowercase letters indicate significant differences (one-way ANOVA, *P* < 0.05).


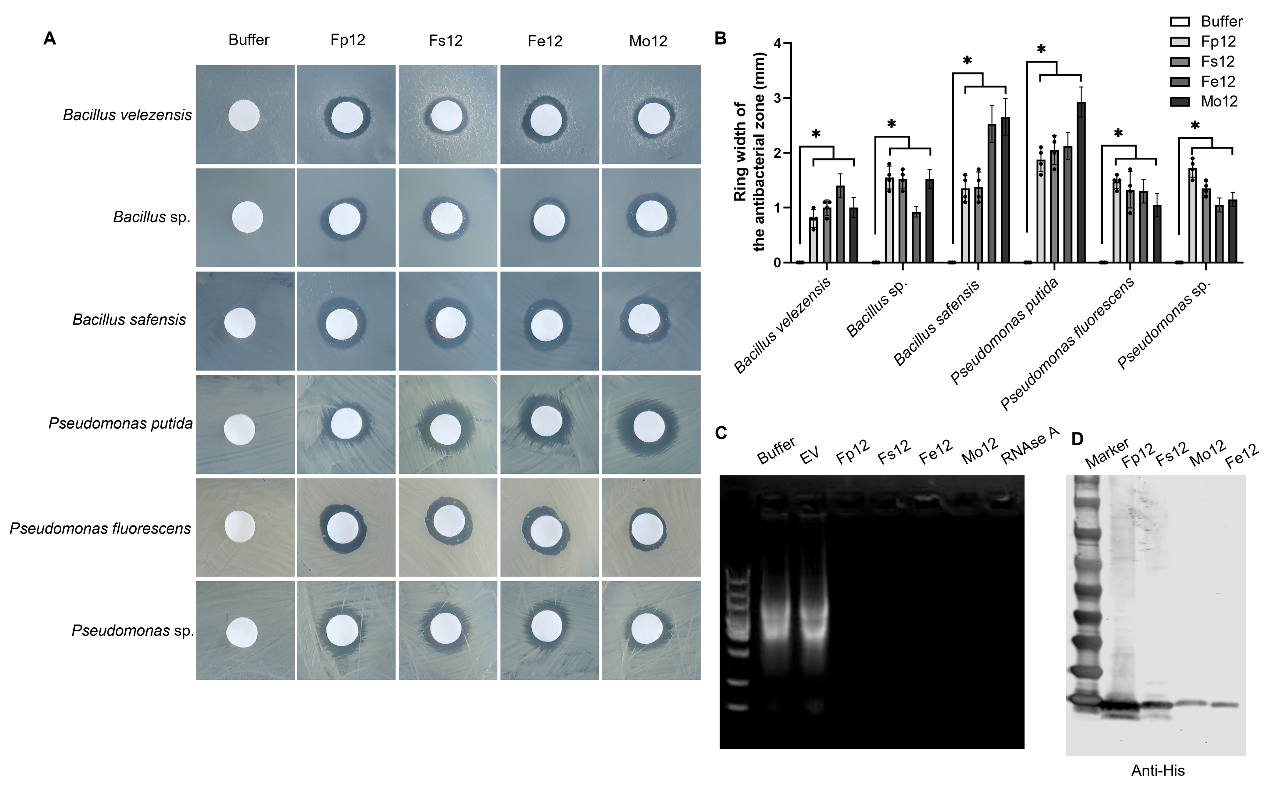


**Figure S9. Antimicrobial and RNase activities of Fg12 homologs.** A) Disk diffusion assay of Fg12 homologs (Fs12, Fp12, Fe12, Mo12; 10 µM) against soybean-associated bacteria. Bacterial plates prepared with OD_600_ = 0.05. Image captured 16 h post-inoculation. B) Antibacterial zone width measured at 16 h. Data represent mean ± SD (N = 4). The asterisk represents significant differences (*P* < 0.05). C) RNase activity of recombinant Fg12 homologs (1 µM) incubated with soybean root RNA at 25°C for 30 min. RNase A (positive control), buffer, and GST (EV control; 1 µM) included. D) Immunoblot of *E. coli*-produced Fg12 homologs detected using anti-his antibody.


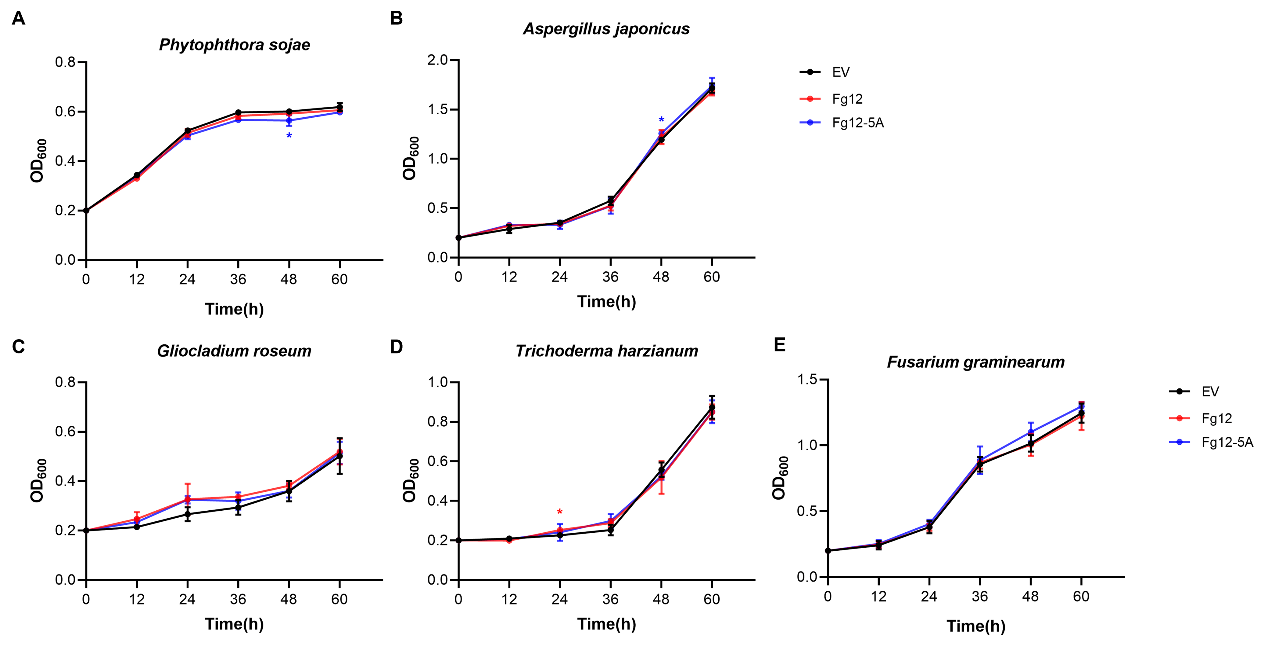


**Figure S10. Fg12 lacks antifungal activity against soybean-associated fungi and *Phytophthora sojae*.** Spores were harvested from PDA and V8 agar plates, diluted, and mixed with protein solution (10µM) in liquid PDB or V8 culture medium. Recombinant Fg12 protein, EV (glutathione S-transferase, GST protein) and the Fg12 enzyme mutant Fg12-5A protein produced by *E. coli* was used. Graphs display the average OD_600_ of three biological replicates ± SD (unpaired t-test, N = 3). The asterisk represents significant differences (*P* < 0.05).


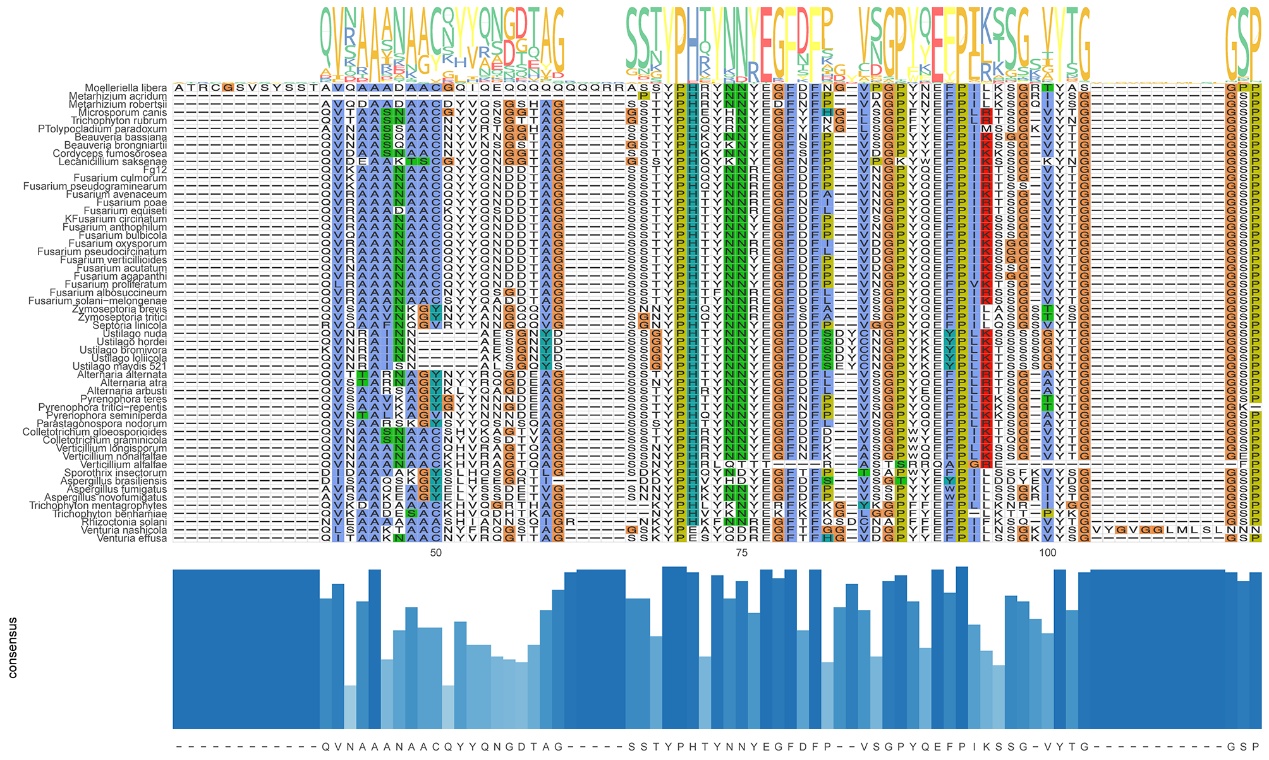


**Figure S11. Alignment of RNase domains of 56 homologous protein sequences.** Alignment of 56 homologous protein sequences revealed conserved regions among these proteins. For each position, the overall height of the stack indicates the sequence conservation at that position, while the height of symbols within the stack indicates the relative frequency of each amino acid at that position. The column at the bottom of the graph represents the conservation of different sites.


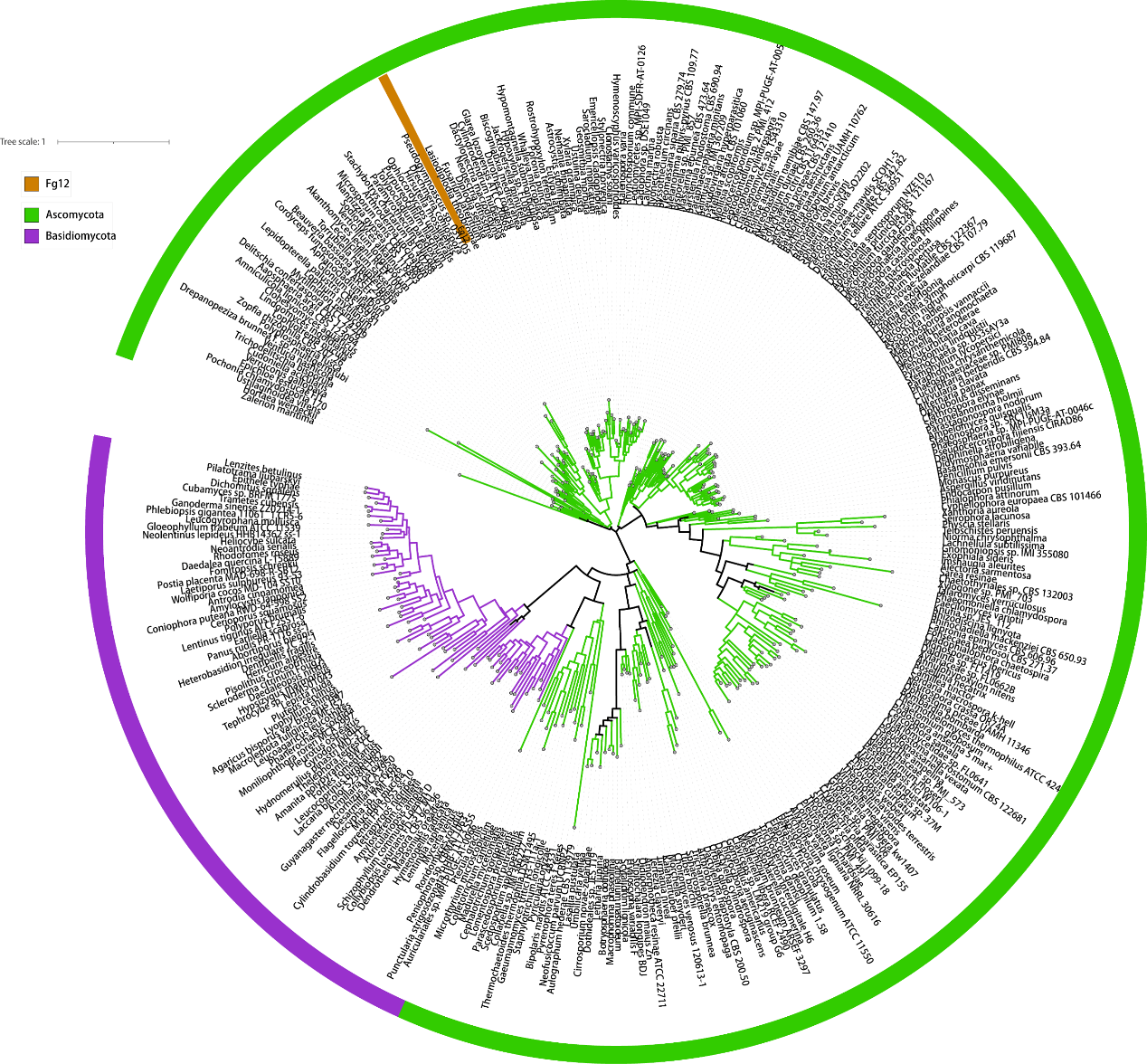


**Figure S12. Fg12 is widely distributed among fungal lineages of Ascomycota and Basidiomycota.** A phylogenetic tree of selected Fg12 homologs reconstructed by RAxML is shown. For these RNase families, we performed multiple sequence alignment and maximum likelihood (ML) tree estimation. Sequence alignment was performed in MAFFT with default settings. TrimAl was used to trim the alignment to eliminate poorly aligned regions. The species tree was estimated in RAxML using the standard algorithm. Visualization of the tree was performed with iTOL.


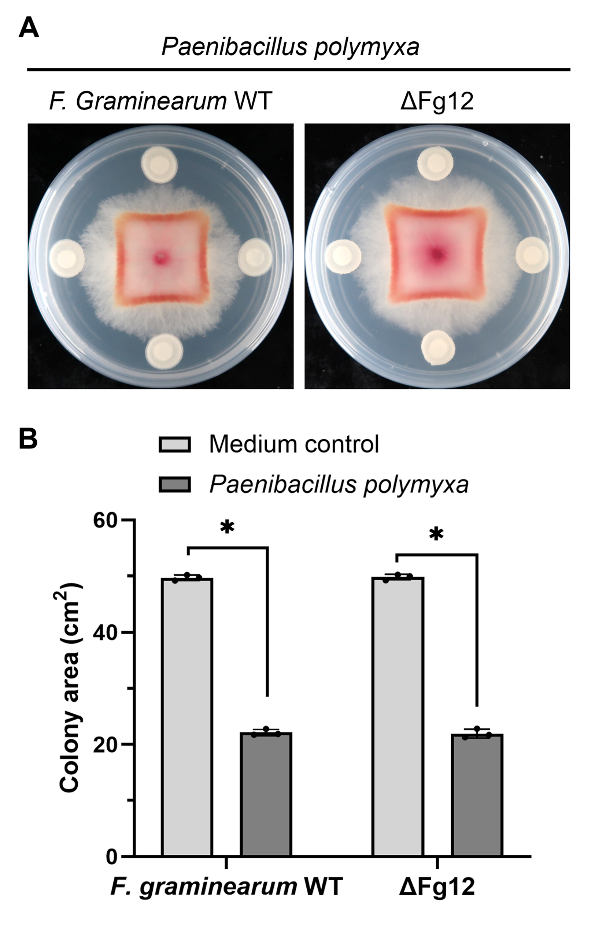


**Figure S13. *Paenibacillus polymyxa antagonizes Fusarium graminearum.*** A) Dual-culture assay: *F. graminearum* PDA plugs (center) were co-cultured with *P. polymyxa*-immersed filter paper (peripheral) or 10 mM MgSO_4_ buffer controls. Image captured after 4 days at 25°C. B) Fungal colony area of *F. graminearum* WT or ΔFg12 after confrontation with *P. polymyxa*. Data represent mean ± SD (unpaired t-test, N = 3). Asterisks denote significance with control (*P* < 0.05).


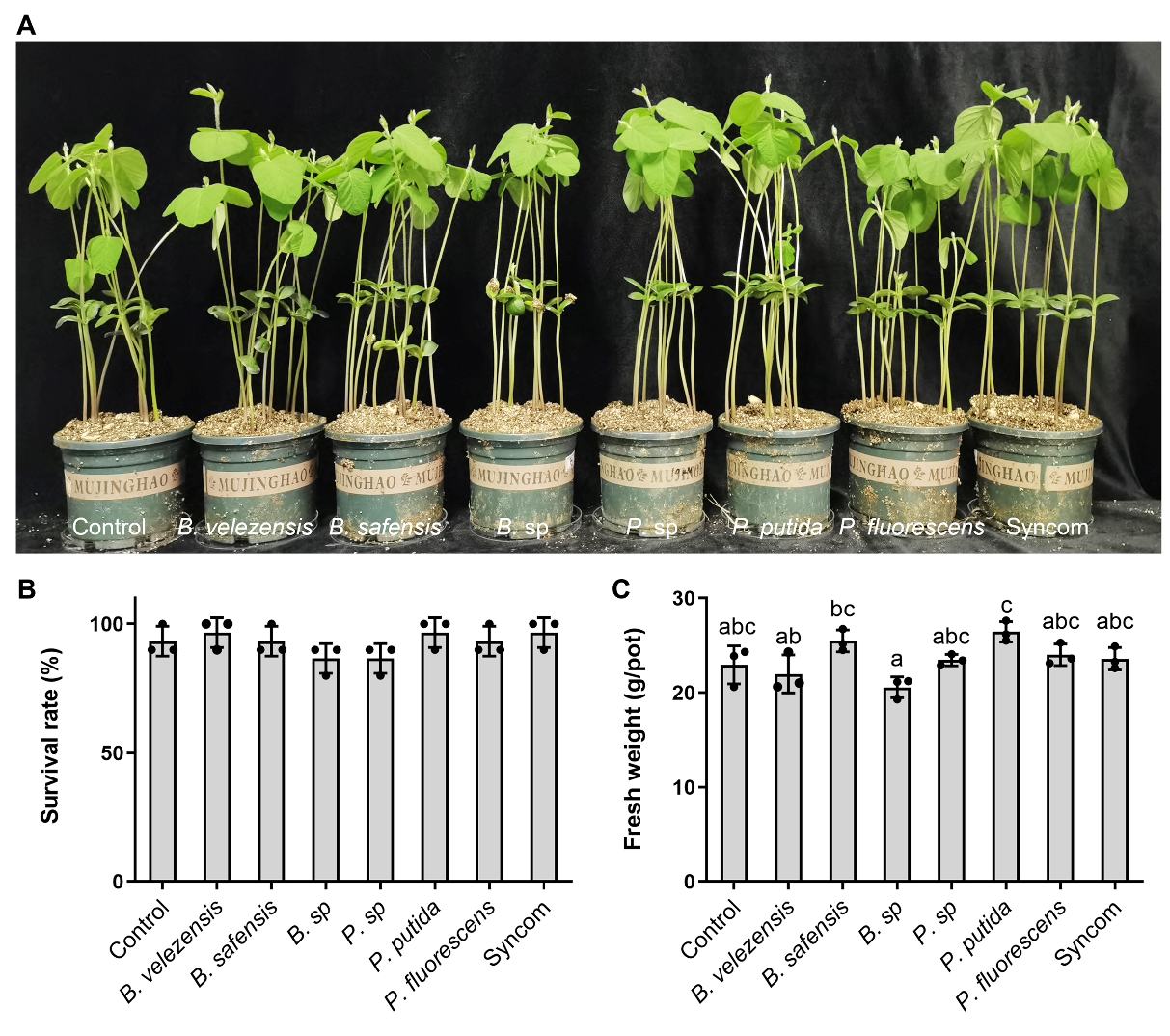


**Figure S14. Plant-associated *Bacillus* and *Pseudomonas* do not impair soybean growth.** A) Soybean phenotypes at 14 d post-inoculation (dpi). HeFeng47 soybeans grown in sterilized soil-vermiculite (7:3) mix. 10 mL suspensions (OD_600_ = 0.1) of plant-associated *Bacillus* or *Pseudomonas* strains applied to pots (10 seeds/pot). Three biological replicates. B) Survival rates across treatments. C) Fresh biomass. Data represent mean ± SD (N = 3). Different lowercase letters indicate significant differences (one-way ANOVA, *P* < 0.05).


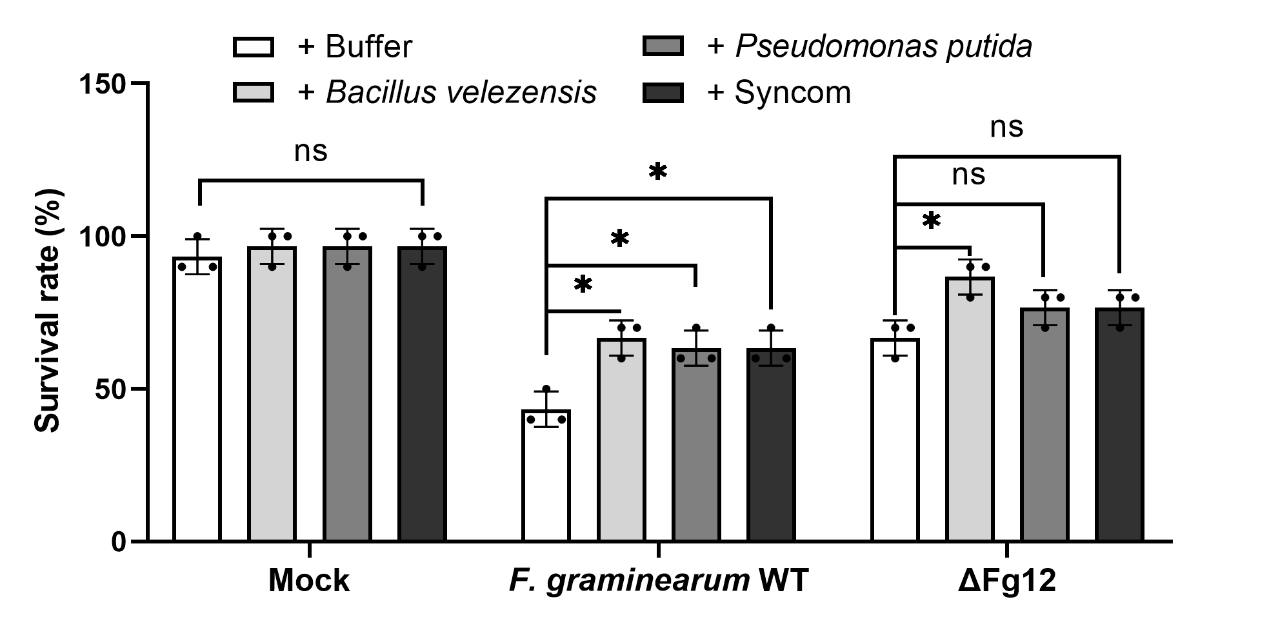


**Figure S15. *Bacillus and Pseudomonas* improve soybean survival rates upon *Fusarium* infection.** Survival rate of soybean plants grown in soils measured at 14 days post-sowing (unpaired t-test, N =3). The asterisk represents significant differences (*P* < 0.05).


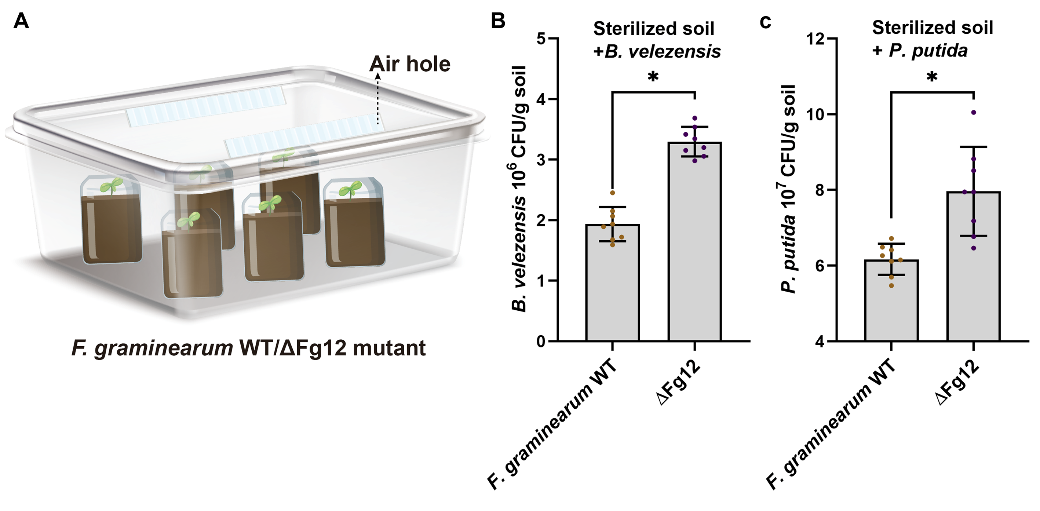


**Figure S16. Fg12 inhibits *Bacillus velezensis* and *Pseudomonas putida* in the soybean rhizosphere.** A) Schematic diagram of the gnotobiotic plant growth system. B, C) Colony-forming unit (CFU) counts of *B. velezensis* and *P. putida* per gram of rhizosphere soil (unpaired t-test, N = 8). Asterisks denote statistically significant differences (*P* < 0.05).


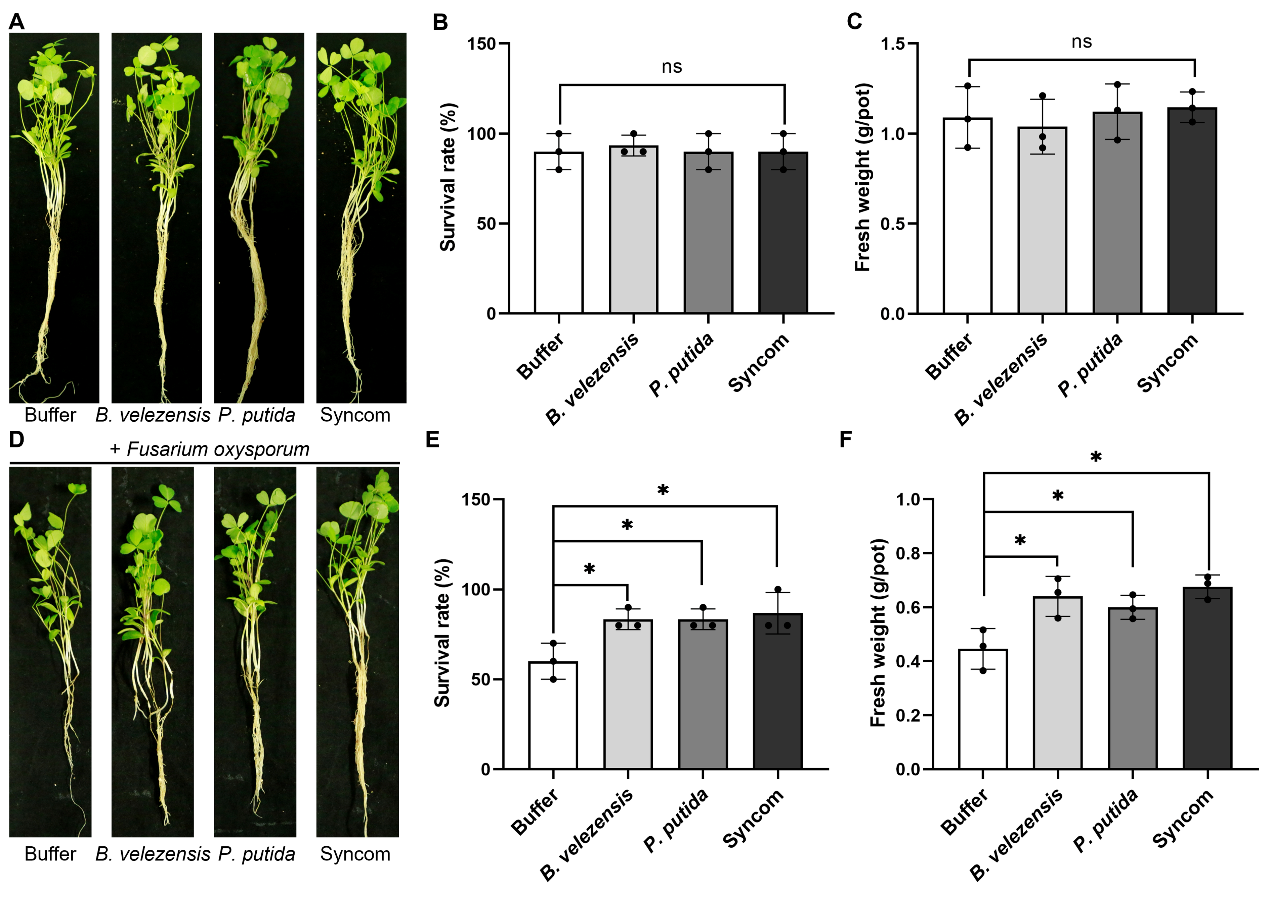


**Figure S17. *Bacillus* and *Pseudomonas* improve alfalfa survival rates upon *Fusarium* infection.** A, D) Phenotypes of alfalfa plants 14 days post-inoculation with *F. oxysporum* or mock-treated controls (PDA medium). Surface-sterilized seeds were cultivated in soil (30% sterilized vermiculite) mixed with *F. oxysporum* mycelium. Each pot received 10 mL of bacterial suspensions (OD_600_=0.1) or 10 mM MgSO₄ (non-inoculated control). For syncom inoculation, overnight cultures of six bacterial strains were resuspended in 10 mM MgSO₄, adjusted to equal OD_600_, pooled in equal volumes, and diluted to OD_600_=0.1. Each pot contained 10 seeds with three independent replicates. B, E) Survival rates of alfalfa plants 14 days post-sowing (unpaired t-test, N = 3). C, F) Fresh biomass of alfalfa plants 14 days post-sowing (unpaired t-test, N = 3). Asterisks indicate statistically significant differences (*P* < 0.05).


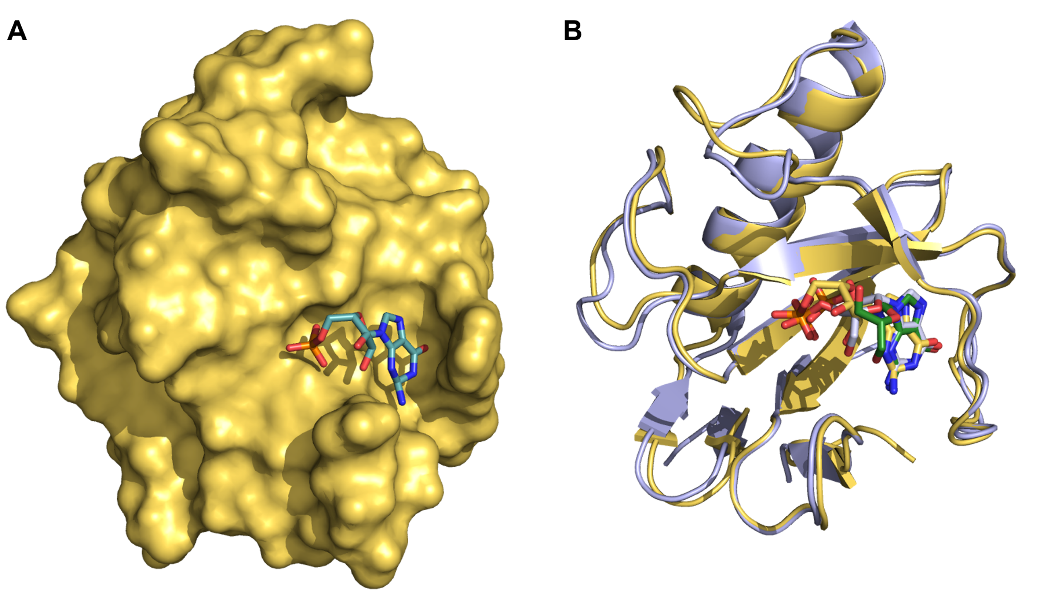


**Figure S18. Interactions of GMP and its isomeride with RNases.** A) The binding patterns of Fg12 and 5’-GMP by the prediction of Molecular Operating Environment (MOE). The ligand–protein interaction behaviors were estimated based on the docking score function, as implemented in the MOE package. The structure of Fg12 is shown in yellow, the compound is 5’-GMP. B) Comparison of binding patterns of Fg12-GMP with reported RNase-GMP. The structure of Fg12 is shown in yellow, and the structure of RNase from *Aspergillus oryzae* is shown in blueviolet (PDB ID: 1RLS). The yellow compound is 5’-GMP, light purple is 3’-GMP, and green is 2’-GMP.


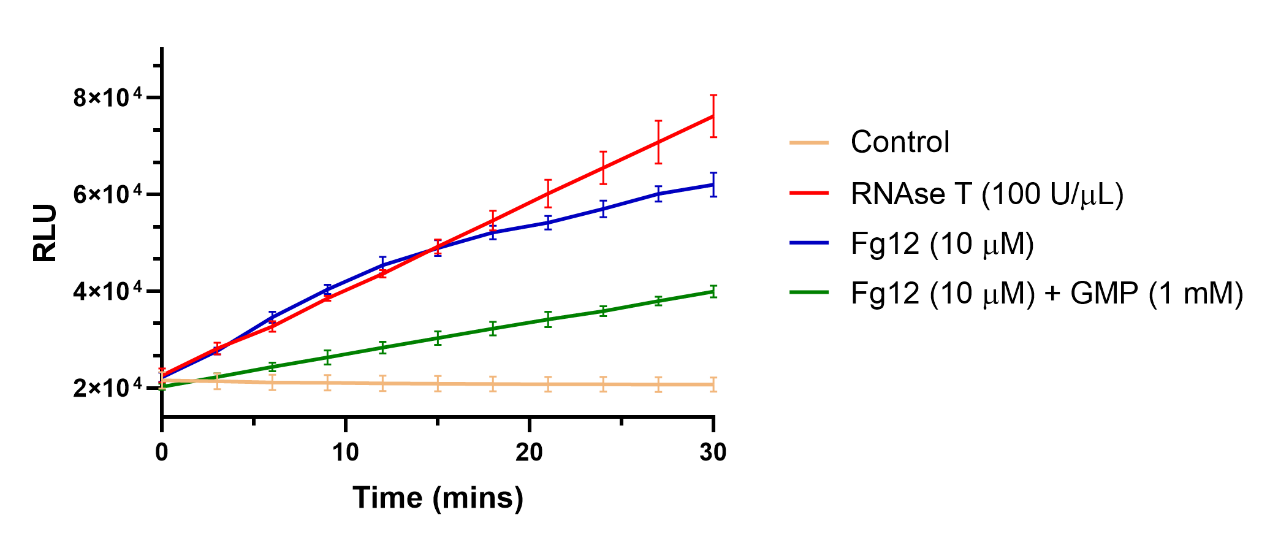


**Figure S19. GMP suppresses RNase activity of Fg12.** RNase activity assays of Fg12 (10 μM recombinant protein expressed in *E. coli*) with or without 1 mM GMP. Protein buffer (negative control) and RNase T (positive control) were included. Activity was quantified at 3-minute intervals for 30 minutes. Data are derived from three independent biological replicates.


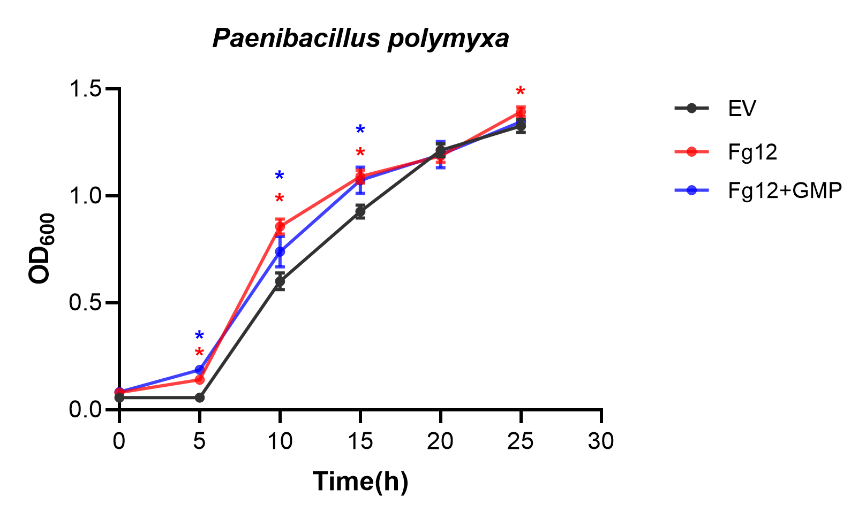


**Figure S20. GMP does not alter the in vitro growth of soybean-associated *Paenibacillus polymyxa***. Recombinant Fg12 protein (10 μM) produced by *E. coli* was used, EV (glutathione S-transferase, GST protein, 10 μM) protein produced by *E. coli* were used as controls. 1 mM GMP was used in this experiment. Growth curves show average OD_600_ values of three biological replicates ± SD. Asterisks indicate statistically significant differences (*P* < 0.05).


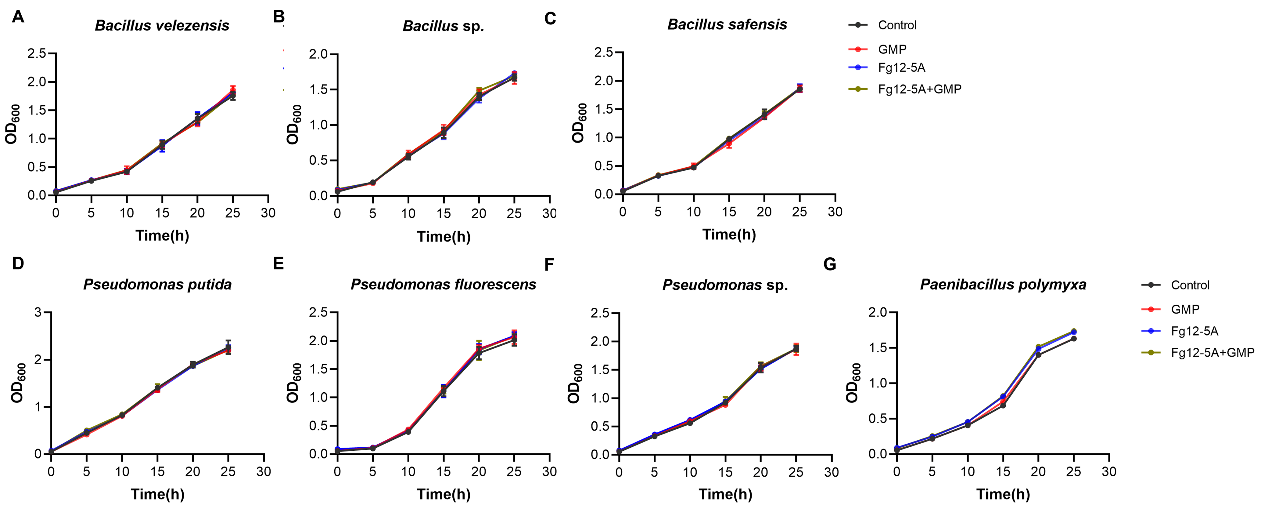


**Figure S21. GMP does not alter the in vitro growth of soybean-associated bacteria.** Growth curves of bacterial cultures supplemented with 1 mM GMP show average OD_600_ values from three biological replicates ± SD.


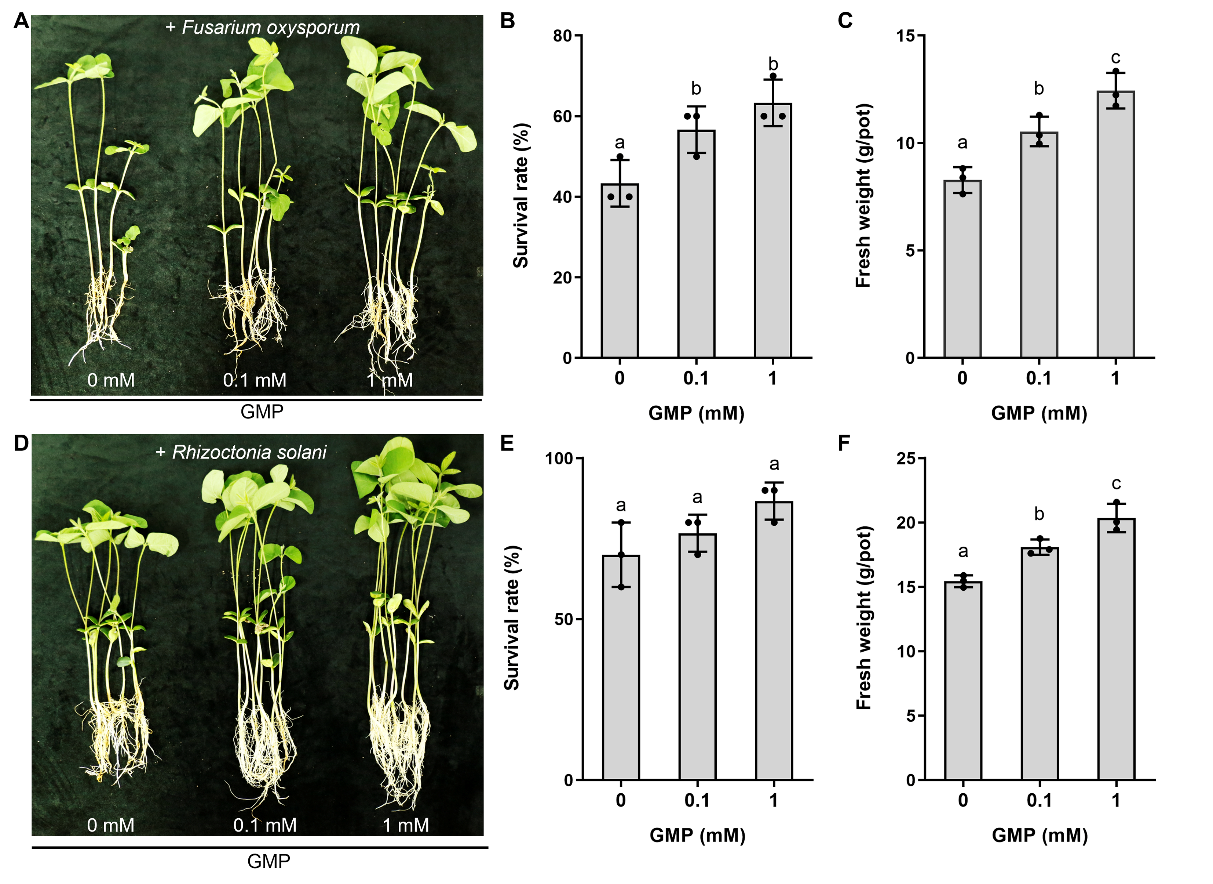


**Figure S22. GMP alleviates fungal infection symptoms in soybean.** A, D) Phenotypes of soybean plants (cultivar Hefeng47) 14 days post-inoculation with *Fusarium oxysporum* (A) or *Rhizoctonia solani* (D). Surface-sterilized seeds were cultivated in soil (30% sterilized vermiculite) containing fungal mycelium, followed by treatment with 10 mL of GMP solutions at varying concentrations. Each pot contained 10 seeds with three independent replicates. B, E) Survival rates of soybean plants 14 days post-sowing. Different lowercase letters above bars indicate statistically significant differences (one-way ANOVA, *P* < 0.05; N = 3). C, F) Fresh biomass of soybean plants 14 days post-sowing. Significant differences are denoted as in B and E.


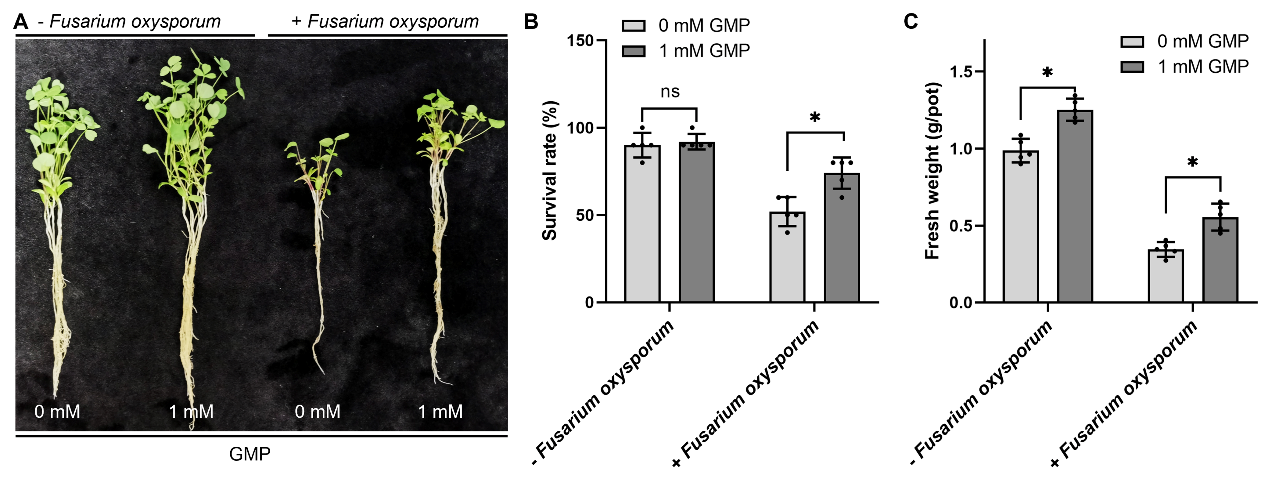


**Figure S23. GMP alleviates *Fusarium* infection symptoms in alfalfa.** A) Phenotypes of alfalfa plants (cultivar ‘Zhongmu No. 1’) 12 days post-inoculation with *Fusarium oxysporum*. Surface-sterilized seeds were cultivated in soil (30% sterilized vermiculite) amended with *F. oxysporum* mycelium, followed by application of 10 mL GMP solutions at varying concentrations. Each pot contained 10 seeds with five independent replicates. B) Survival rates and C) fresh biomass of alfalfa plants 12 days post-sowing. Asterisks indicate statistically significant differences (unpaired t-test, *P* < 0.05; N = 5).


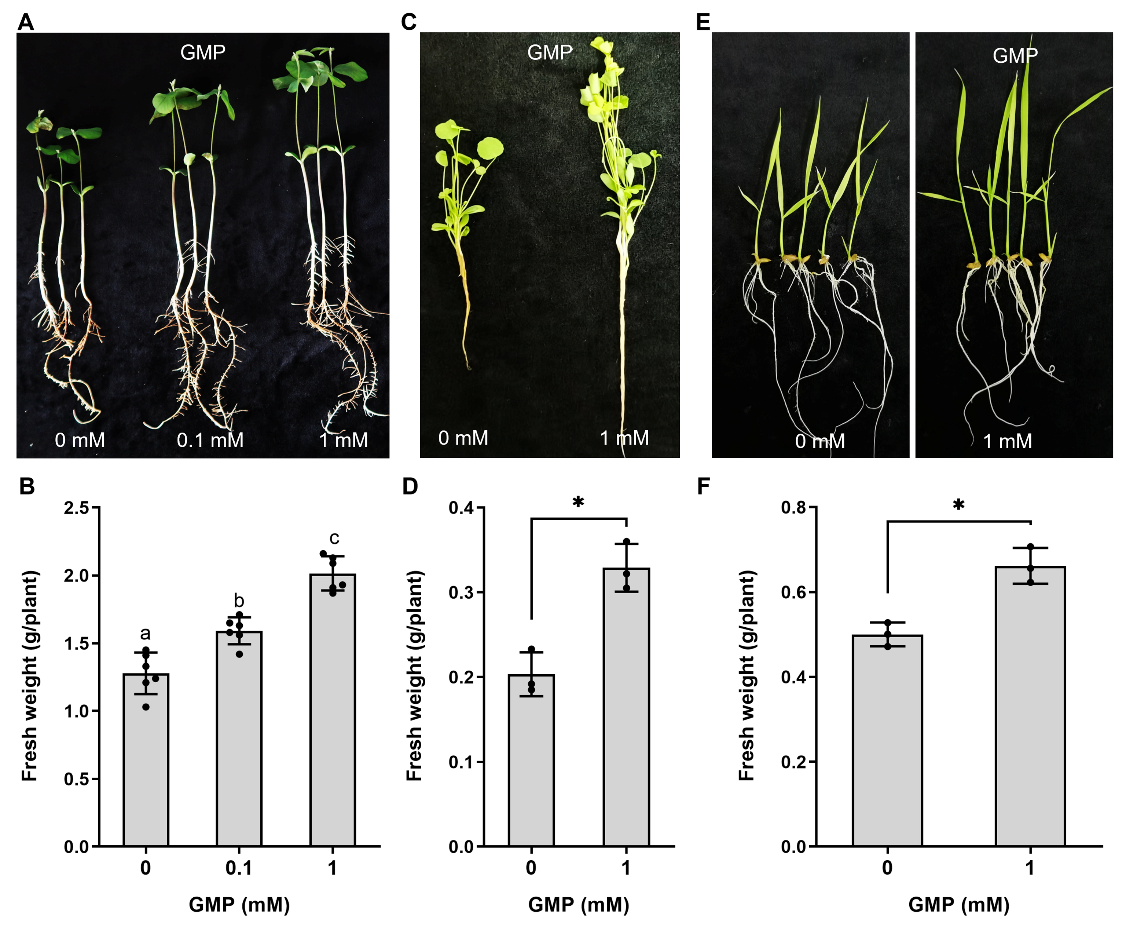


**Figure S24. GMP promotes growth of multiple plant species.** A, C, E) Representative images of soybean (cv. Hefeng47), alfalfa (cv. Zhongmu No. 1), and rice (cv. Nipponbare) plants after 14-day growth under different treatments. Surface-sterilized seeds were cultivated in hydroponic solutions supplemented with varying GMP concentrations. B) Fresh biomass of soybean plants. Data represent mean ± SD of six plants pooled from three experiments. Different lowercase letters indicate significant differences (one-way ANOVA, *P* < 0.05; N = 6). D, F) Fresh biomass of alfalfa (D) and rice (F) plants. Data are mean ± SD from three independent experiments. Asterisks denote statistically significant differences (unpaired t-test, *P* < 0.05; N = 3).


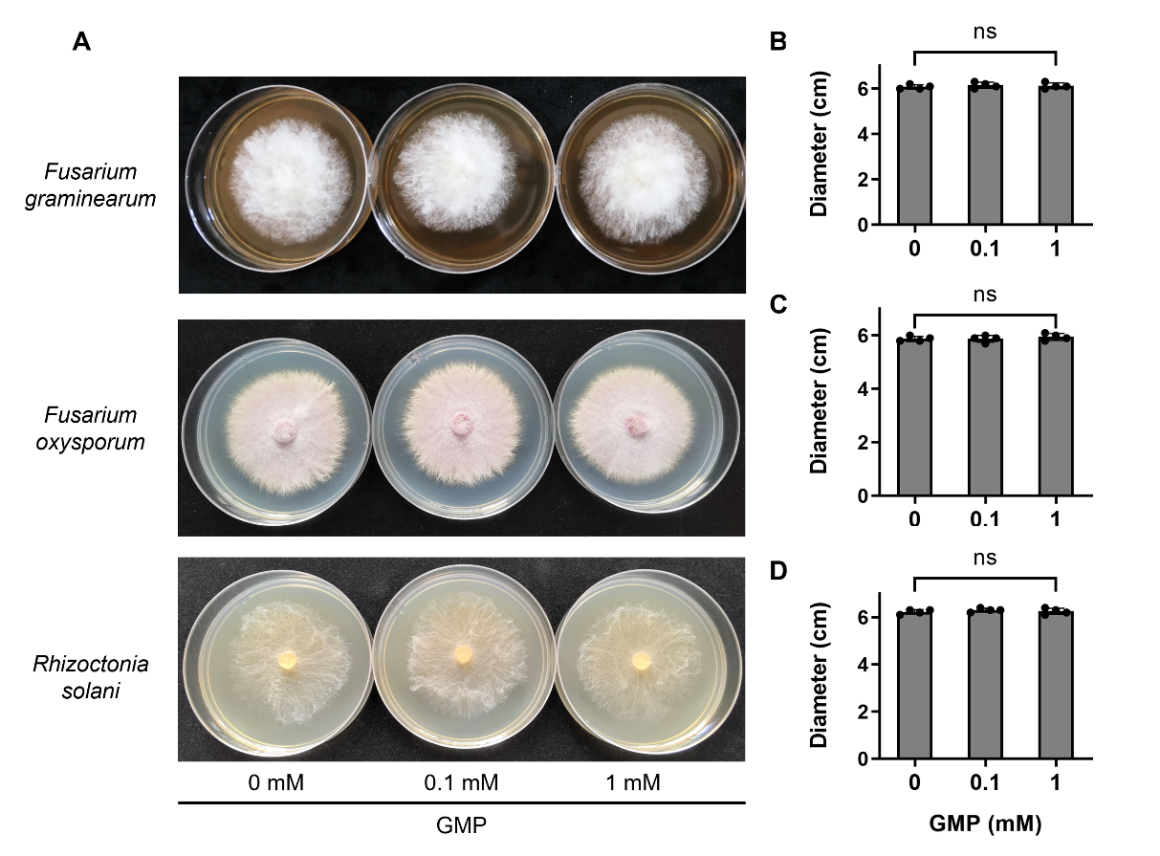


**Figure S25. GMP displays no adverse effects on the growth of *Fusarium graminearum*, *F. oxysporum*, and *Rhizoctonia solani*.** A) Morphology of *F. graminearum*, *F. oxysporum*, and *R. solani* cultured on potato dextrose agar (PDA) plates containing varying GMP concentrations at 25°C for 4 days (*Fusarium spp*.) or 2 days (*R. solani*) under dark conditions. B-D) Colony diameters of *F. graminearum* (B), *F. oxysporum* (C), and *R. solani* (D) on PDA plates. Data represent mean ± SD of four biological replicates (one-way ANOVA, *P* < 0.05; N = 4).
